# BAYESIAN VARIABLE SELECTION IN A COX PROPORTIONAL HAZARDS MODEL WITH THE “SUM OF SINGLE EFFECTS” PRIOR

**Published:** 2025-06-06

**Authors:** YUNQI YANG, KARL TAYEB, PETER CARBONETTO, XIAOYUAN ZHONG, CAROLE OBER, MATTHEW STEPHENS

**Affiliations:** 1 Committee on Genetics, Genomics and System Biology, University of Chicago, Chicago, IL; 2 Department of Human Genetics, University of Chicago, Chicago, IL; 3 Departments of Statistics and Human Genetics, University of Chicago, Chicago, IL

**Keywords:** time-to-event data, survival analysis, Cox proportional hazards model, Bayesian variable selection in regression, genomics, genome-wide association studies, genetic fine-mapping, UK Biobank

## Abstract

Motivated by genetic fine-mapping applications, we introduce a new approach to Bayesian variable selection regression (BVSR) for time-to-event (TTE) outcomes. This new approach is designed to deal with the specific challenges that arise in genetic fine-mapping, including: the presence of very strong correlations among the covariates, often exceeding 0.99; very large data sets containing potentially thousands of covariates and hundreds of thousands of samples. We accomplish this by extending the “Sum of Single Effects” (SuSiE) method to the Cox proportional hazards (CoxPH) model. We demonstrate the benefits of the new method, “CoxPH-SuSiE”, over existing BVSR methods for TTE outcomes in simulated fine-mapping data sets. We also illustrate CoxPH-SuSiE on real data by fine-mapping asthma loci using data from UK Biobank. This fine-mapping identified 14 asthma risk SNPs in 8 asthma risk loci, among which 6 had strong evidence—a posterior inclusion probability greater than 50%—for being causal. Two of the 6 putatively causal variants are known to be pathogenic, and others lie within a genomic sequence that is known to regulate the expression of *GATA3*.

## 1. Introduction.

With the increasing availability of biobanks and electronic health records, analyzing time-to-event (TTE) phenotypes such as disease age of onset has become more common in genetics. TTE data can yield insight into the genetics of disease development and progression, and enhance our understanding of disease etiology. Research has shown that modeling TTE phenotypes using survival models can be more powerful than modeling binary disease status using logistic models, particularly for events that are more common (Callas, Pastides and Hosmer, 1998; Green and Symons, 1983; Hughey et al., 2019; Staley et al., 2017). Additionally, genome-wide association studies (GWAS) of TTE phenotypes have identified significant loci that are not significant based on case-control status (Bi et al., 2020).

After detecting a genetic association, a common next step is “fine-mapping”, which is the process of narrowing the set of associated genetic variants down to a smaller set of causal candidates (Hutchinson, Asimit and Wallace, 2020; Kote-Jarai et al., 2013; Maller et al., 2012; Schaid, Chen and Larson, 2018). The most widely used fine-mapping approaches frame the fine-mapping as a variable selection problem within a regression model, in which the variables in the regression are the genetic variants (George and McCulloch, 1997; Raftery, Madigan and Hoeting, 1997; Sillanpää and Bhattacharjee, 2005). Fine-mapping is a particularly challenging variable selection problem because of the strong correlations that can exist among genetic variants; pairs of nearby genetic variants can have sample correlations of 0.99 or higher.

The very strong correlations, or “linkage disequilibrium” (LD), among the genetic variants often means that we cannot select variables confidently. For this reason, quantifying uncertainty in variable selection becomes important, and Bayesian approaches to variable selection in regression (BVSR) are particularly good at dealing with this. BVSR methods for fine-mapping typically provide two key quantities: a posterior inclusion probability (PIP) for each variable, which gives the probability that this variable is a causal variable (which we use as shorthand for a variable with a truly non-zero regression coefficient); and “Credible Sets” (CSs) of variables, which are defined as sets of variables that have a high probability (e.g., 0.95 or greater) of containing at least one causal variable (Maller et al., 2012; Wang et al., 2020).

Several BVSR methods and software for fine-mapping quantitative traits are available, including Benner et al. (2016); Chen et al. (2015); Hormozdiari et al. (2014); Kichaev et al. (2014); Lee et al. (2018); Li and Kellis (2016); Maller et al. (2012); Wallace et al. (2015); Wang et al. (2020); Wen et al. (2016); Yang et al. (2012); Zou et al. (2022). All these methods are based on linear regression models with Gaussian errors. For analyzing TTE phenotypes using survival models, however, the options are limited, and none of the available methods provide CSs. Newcombe et al. (2017) propose methods for sparse Bayesian Weibull regression with a normal prior for the non-zero effect variables, and they performed posterior inferences using a reversible jump Markov chain Monte Carlo (MCMC) algorithm. Nikooienejad, Wang and Johnson (2020) developed a BVSR method using the Cox proportional hazards (CoxPH) model (Cox, 1972), which is the most widely used model for TTE outcomes. They used a nonlocal prior for the non-zero regression coefficients and a stochastic search algorithm for inference. More recently, Komodromos et al. (2022) introduced a sparse variational Bayes approach for the CoxPH model, with a Laplace prior for the non-zero effect variables. While all of these methods provide PIPs, none of them provide the CSs. Further, some of these methods have not been tested on data sets containing strongly correlated variables, leaving open the question of how well they will perform in fine-mapping settings with very strongly correlated genetic variants.

Here we introduce a new BVSR method for TTE outcomes that is designed to address these issues: it provides both CSs and PIPs, and it is specifically designed to cope with highly correlated covariates. This new method combines the CoxPH model with the “Sum of Single Effects” (SuSiE) method introduced in Wang et al. (2020). SuSiE was specifically designed to provide CSs and PIPs, and to deal with the very high correlations that occur in fine-mapping applications. It is also fast, and able to deal with large data sets containing thousands of covariates. Our new fine-mapping method for TTE outcomes, “CoxPH-SuSiE”, extends the advantages of SuSiE to the CoxPH regression model. Further, this approach of combining SuSiE with a single-variable regression model is quite general, and could be easily adapted to other regression models such as logistic regression or Poisson regression. R code implementing the new methods is available online at https://github.com/yunqiyang0215/survival-susie/ (see also Yang et al. 2025).

### 1.1. Organization of the paper.

The remainder of the paper is organized as follows. Section 2 presents necessary background. Section 3 describes our new approach, CoxPH-SuSiE. Section 4 numerically compares the accuracy of different approaches for computing the Bayes factors for CoxPH regression models, which is a key step in CoxPH-SuSiE. Then Section 5 assesses the performance of CoxPH-SuSiE and other BVSR methods in simulated fine-mapping data sets with a TTE outcome. Section 6 presents an application of CoxPH-SuSiE to fine-mapping asthma loci using data from UK Biobank. Finally, Section 7 discusses benefits and practical limitations of CoxPH-SuSiE, and possible future directions.

## 2. The CoxPH and SuSiE models.

Our new methods involve combining two essential building blocks: the Cox proportional hazards (CoxPH) model for survival analysis (Cox, 1972), and the SuSiE prior model for BVSR (Wang et al., 2020), which we now describe.

### 2.1. The CoxPH model.

The CoxPH model is a model for time-to-event (TTE) data in a prospective cohort study where the measured data on each individual i is *either* the time $T_{i}$ at which a particular event (e.g., disease diagnosis) occurs *or* the time $C_{i}$ at which the individual left the study without experiencing the event, known as the “censoring” time. For notational convenience we assume that every individual has both an event time and a censoring time, but we only observe whichever occurs first. Thus the outcomes for a cohort of n individuals are denoted as (y,δ), where $y=\left(y_{1},\ldots ,y_{n}\right)$, $\delta =\left(\delta_{1},\ldots ,\delta_{n}\right)$, $y_{i}=\text{min}\left(T_{i},C_{i}\right)$, and $\delta_{i}=\text{I}\left(T_{i}\leq C_{i}\right)$ is 1 when individual i was uncensored, and zero otherwise. The total number of observed events is $K:=\sum_{i=1}^{n}\delta_{i}$, and the ratio $\frac{n-K}{n}$ is referred to as the “censoring rate”.

CoxPH regression models the relationship between the event times $T_{i}$ and covariates, $x_{i}={\left(x_{i1},\ldots ,x_{ip}\right)}^{⊤}$, through the *hazard function* λ, defined as

(1) $${\text{λ}}_{i}(t)=\underset{\text{Δ}\to 0}{\text{lim}}\frac{1}{\text{Δ}}\text{Pr}\left(t\leq T<t+\text{Δ}|T\geq t,x_{i}\right).$$

Specifically, CoxPH assumes

(2) $${\text{λ}}_{i}(t)={\text{λ}}_{0}(t)\;\text{exp}\left(b^{⊤}x_{i}\right),$$

where $b={\left(b_{1},\ldots ,b_{p}\right)}^{⊤}$ is a vector of coefficients to be estimated, and ${\text{λ}}_{0}(t)$ is some (unknown) baseline hazard function. Cox (1972) proposed to estimate b by maximizing a conditional likelihood, also known as the *partial likelihood*. The partial likelihood has the advantage that it does not depend on the unknown baseline hazard, ${\text{λ}}_{0}(t)$. Additionally, the partial likelihood can deal with censoring (Cox, 1975).

### 2.2. The SuSiE prior.

The SuSiE model was introduced in Wang et al. (2020) in the context of linear regression. The SuSiE model combines the linear regression likelihood with a prior on the regression coefficients b that we call the “SuSiE prior”. The SuSiE prior constructs the regression coefficients b as the sum of L “single effect vectors”, $b_{l}$, each of which has exactly one non-zero element, whose value is $b_{l}$:

(3) $$\begin{array}{l} b=\sum_{l=1}^{L}b_{l}\\ b_{l}=b_{l}\gamma_{l}\\ b_{l}∼N\left(0,\sigma_{0l}^{2}\right)\\ \gamma_{l}∼\text{Multinom}(1,\pi ), \end{array}$$

where $N\left(\mu ,\sigma^{2}\right)$ denotes the univariate normal distribution with mean μ and variance $\sigma^{2}$; Multinom (n,π) denotes the multinomial distribution for n multinomial trials with probabilities $\pi =\left(\pi_{1},\ldots ,\pi_{p}\right)$; and $\gamma_{l}\in {\left\{0,1\right\}}^{p}$ is a binary vector indicating which element of $b_{l}$ is non-zero. Like Wang et al. (2020), we assume $\pi_{1}=\cdots =\pi_{p}=\frac{1}{p}$, so all covariates are equally likely to have non-zero coefficients, although other values for π could be used.

Because each $b_{l}$ has exactly one non-zero entry, the SuSiE prior ensures that b has at most L non-zeros. In fine-mapping settings, L is typically small (e.g., L=10), and so the SuSiE prior is sparse. For now, we assume L and the prior variances $\sigma_{0l}^{2}$ are pre-specified; later, we discuss estimating these hyperparameters.

## 3. The CoxPH-SuSiE model.

CoxPH-SuSiE performs Bayesian variable selection for survival analysis by combining the CoxPH partial likelihood (Section 2.1) with the SuSiE prior (Section 2.2). Like all other Bayesian variable selection methods, the inferences in CoxPH-SuSiE are based on Bayes’ Theorem, except that the likelihood in Bayes’ Theorem is replaced with the partial likelihood. Kalbfleisch (1978) showed that using the partial likelihood implicitly approximates standard Bayesian inferences by assuming a limiting gamma process prior for the baseline hazard ${\text{λ}}_{0}(t)$; see also Ibrahim, Chen and Sinha (2001); Sinha, Ibrahim and Chen (2003). We refer to the model that combines the CoxPH partial likelihood with the SuSiE prior as the “CoxPH-SuSiE model”.

In the following sections, we derive the core underlying posterior computations for CoxPH-SuSiE as well as an efficient algorithm for fitting the CoxPH-SuSiE model.

### 3.1. Bayesian CoxPH regression for a single variable.

To build toward the CoxPH-SuSiE model, we first consider a much simpler CoxPH regression model with a *single* covariate, $x={\left(x_{1},\ldots ,x_{n}\right)}^{⊤}$, and a *single* regression coefficient, b. In this model, we assume a normal prior for b, and for later use we allow for a fixed offset, $c={\left(c_{1},\ldots ,c_{n}\right)}^{⊤}$:

(4) $$\begin{array}{l} {\text{λ}}_{i}(t)={\text{λ}}_{0}(t)\;\text{exp}\left(bx_{i}+c_{i}\right)\\ b∼N\left(0,\sigma_{0}^{2}\right). \end{array}$$

We call this the “single-variable CoxPH regression model”.

Let ℓ(b;x,c) denote the Cox partial likelihood for this model. (This likelihood, as well as other quantities, also depend on y, δ , but to keep notation light we suppress this dependence.) Let $\hat{b}(x,c):=\text{argma}{\text{x}}_{b}\ell (b;x,c)$ denote the maximum partial likelihood estimate, and s(x,c) denote its (estimated) standard error. For notational simplicity we often write $\hat{b}$ and s, suppressing their explicit dependence on x and c. Both $\hat{b}$ and s are easily obtained from standard software, such as the coxph() function in the survival R package (Therneau and Grambsch, 2000).

The posterior distribution for b under this model is

(5) $$p\left(b|x,y,\delta ,c,\sigma_{0}^{2}\right)\propto \ell (b;x,c)p\left(b|\sigma_{0}^{2}\right),$$

and the Bayes Factor (BF) (Kass and Raftery, 1995) comparing this model to the null model (b=0) is

(6) $$\text{BF}\left(x,c,\sigma_{0}^{2}\right):=\frac{\int \ell (b;x,c)p\left(b|\sigma_{0}^{2}\right)db}{\ell (0;x,c)}.$$

(The null likelihood ℓ(0;x,c) does not depend on x, but x is included in the notation for consistency.)

Neither the posterior distribution (5) nor the BF (6) have a closed-form solution. While it would be possible to approximate these quantities using numerical integration methods, here we take a simpler (and potentially faster) approach by taking a quadratic approximation to the partial log-likelihood,

(7) $$\text{log}\;\ell (b;x,c)\approx \text{log}\;\ell (\hat{b};x,c)-\frac{{\left(b-\hat{b}\right)}^{2}}{2s^{2}},$$

which yields the following approximation to the likelihood:

(8) $$\ell (b;x,c)\approx \hat{\ell }(b;x,c):=\ell (\hat{b};x,c)\;\text{exp}\left\{-\frac{1}{2s^{2}}{\left(b-\hat{b}\right)}^{2}\right\}.$$

This approximation yields a Gaussian posterior distribution for b,

(9) $$b|x,y,\delta ,c,\sigma_{0}^{2}∼N\left(\mu_{1},\sigma_{1}^{2}\right),$$

where

(10) $$\sigma_{1}^{2}\left(x,c,\sigma_{0}^{2}\right):=\frac{1}{1/s^{2}+1/\sigma_{0}^{2}}$$

(11) $$\mu_{1}\left(x,c,\sigma_{0}^{2}\right):=\frac{\sigma_{1}^{2}\left(x,c,\sigma_{0}^{2}\right)}{s^{2}}\times \hat{b}.$$

Letting $z:=\hat{b}/s$, the corresponding approximate BF is

(12) $$\begin{array}{l} \hat{\text{BF}}\left(x,c,\sigma_{0}^{2}\right):=\frac{\int \hat{\ell }(b;x,c)p\left(b|\sigma_{0}^{2}\right)db}{\ell (0;x,c)}\\ =\text{ABF}\left(x,c,\sigma_{0}^{2}\right)\times \text{exp}\left(\frac{-z^{2}}{2}\right)\times \frac{\ell (\hat{b};x,c)}{\ell (0;x,c)}, \end{array}$$

where $\text{ABF}\left(x,c,\sigma_{0}^{2}\right)$ denotes the “asymptotic Bayes factor” (Wakefield, 2009),

(13) $$\text{ABF}\left(x,c,\sigma_{0}^{2}\right)=\sqrt{\frac{s^{2}}{\sigma_{0}^{2}+s^{2}}}\times \text{exp}\left(\frac{z^{2}}{2}\times \frac{\sigma_{0}^{2}}{\sigma_{0}^{2}+s^{2}}\right).$$

The approximation (12) is a variant of the standard Laplace approximation to the Bayes Factor (Kass and Raftery, 1995). Comparisons below (Section 4) show that this Laplace approximation to the Bayes factor (“Laplace BF”) is accurate, and can be substantially more accurate than the ABF approximation (13).

### 3.2. Single effect regression for the Bayesian CoxPH model.

Wang et al. (2020) defined a “single effect regression” (SER) as a multiple regression model in which *exactly one of* p *covariates* has a non-zero coefficient in the model. (The SER model is a special case of the SuSiE model, with L=1.) The analogous SER model for CoxPH regression with fixed offset c is

(14) $$\begin{array}{rr} {\text{λ}}_{i}(t) & ={\text{λ}}_{0}(t)\;\text{exp}\left(b^{⊤}x_{i}+c_{i}\right)\\ b & =b\gamma \\ \gamma & ∼\text{Multinom}(1,\pi )\\ b & ∼N\left(0,\sigma_{0}^{2}\right). \end{array}$$

In the following, we assume the data for the p covariates are stored as an n×p matrix X, in which $x_{i}$ denotes the ith row of X and $x_{\cdot j}$ denotes the jth column of X.

Under this model, the posterior distribution of γ is

(15) $$\begin{array}{l} \gamma \left|X,y,\delta \right.∼\text{Multinom}\left(1,\alpha \right)\\ \text{Pr}\left(\gamma_{j}=1|X,y,\delta \right)=\frac{\pi_{j}BF_{j}}{\sum_{j^{\prime }=1}^{p}\pi_{j^{\prime }}\text{B}{\text{F}}_{j^{\prime }}}, \end{array}$$

where $\text{B}{\text{F}}_{j}:=\text{BF}\left(x_{\cdot j},c,\sigma_{0}^{2}\right)$ is the Bayes factor (6) that compares the CoxPH model with the jth column of X as the covariate vs. the null model. In practice, we compute these posterior probabilities by replacing the exact Bayes factors with their approximations,

(16) $$\text{Pr}\left(\gamma_{j}=1|X,y,\delta \right)\approx \alpha_{j}:=\frac{\pi_{j}{\hat{\text{BF}}}_{j}}{\sum_{j^{\prime }=1}^{p}\pi_{j^{\prime }}{\hat{\text{BF}}}_{j^{\prime }}},$$

where ${\hat{\text{BF}}}_{j}:=\hat{\text{BF}}\left(x_{\cdot j},c,\sigma_{0}^{2}\right)$; see eq.(12). The corresponding approximate posterior distribution of b given $\gamma_{j}=1$ is normal with mean $\mu_{1j}$ and variance $\sigma_{1j}^{2}$, where

(17) $$\sigma_{1j}^{2}=\sigma_{1}^{2}\left(x_{\cdot j},c,\sigma_{0}^{2}\right)$$

(18) $$\mu_{1j}=\mu_{1}\left(x_{\cdot j},c,\sigma_{0}^{2}\right),$$

using the definitions in (10, 11).

In summary, computing the approximate posterior distribution for the CoxPH SER model on p covariates involves computing three p-vectors, $\alpha =\left(\alpha_{1},\ldots ,\alpha_{p}\right)$, $\mu_{1}=\left(\mu_{11},\ldots ,\mu_{1p}\right)$ and $\sigma_{1}^{2}=\left(\sigma_{11}^{2},\ldots ,\sigma_{1p}^{2}\right)$, whose elements are given by (16–18). To express these posterior computations succinctly, we define the following function:

(19) $$\text{COXPH}-\text{SER}\left(X,y,\delta ,c;\sigma_{0}^{2}\right):=\left(\alpha ,\mu_{1},\sigma_{1}^{2}\right).$$

We note that, given these quantities, the posterior mean of the single effect vector b is easily computed as $\bar{b}=\alpha \circ \mu_{1}$ where “∘” denotes element-wise multiplication.

#### Estimating the prior variance.

The SER hyperparameter $\sigma_{0}^{2}$ can be fixed, or it can be estimated by maximizing the approximate (partial) likelihood, which is given by

(20) $${\hat{\ell }}_{\text{SER}}\left(\sigma_{0}^{2}\right)=\sum_{j=1}^{p}\pi_{j}\int \hat{\ell }(b;x\cdot j,c)p\left(b|\sigma_{0}^{2}\right)db.$$

A simple expectation maximization (EM) algorithm (Dempster, Laird and Rubin, 1977) can be used to maximize ${\hat{\ell }}_{\text{SER}}$. This EM algorithm cycles between computing the posterior quantities (19) at a given $\sigma^{2}$ (this is the E-step), then updating $\sigma_{0}^{2}$ by

(21) $$\sigma_{0}^{2}\leftarrow \sum_{j=1}^{p}\alpha_{j}\left(\mu_{1j}^{2}+\sigma_{1j}^{2}\right)$$

(this is the M-step). See the Appendix for a derivation of this EM algorithm.

### 3.3. CoxPH-SuSiE.

A key feature of the SuSiE model from Wang et al. (2020) is that, given estimates of $b_{1},\ldots ,b_{L-1}$, estimating $b_{L}$ corresponds to fitting a Gaussian SER model with offset $c=\sum_{l=1}^{L-1}Xb_{l}$. As Wang et al. (2020) notes, this suggests an *iterative approach* to fitting a SuSiE model: the idea is to cycle through the L individual SER models, updating the offset and fitting a Gaussian SER model. They called this iterative model fitting procedure “Iterative Bayesian Stepwise Selection” (IBSS). Here we generalize these ideas to fit the CoxPH-SuSiE model, resulting in an iterative algorithm which in each iteration of the algorithm fits a CoxPH-SER model. We call the resulting algorithm “generalized Iterative Bayesian Stepwise Selection” (gIBSS); it is summarized in Algorithm 1.

gIBSS returns approximate posterior distributions for the single effect vectors $b_{l}$. We use these approximate posterior distributions to compute, for each l, a point estimate of $b_{l}$ (we typically use the posterior mean, ${\bar{b}}_{l}$) and a Credible Set (CS). A CS is a set of variables that has a high probability of containing the variable with the non-zero regression coefficient.

#### Definition 3.1 (Wang et al. 2020).

A level-ρ Credible Set is a subset of variables that has probability ≥ρ of containing at least one effect variable (i.e., a variable with non-zero regression coefficient).

Given $\alpha_{l}$, it is straightforward to construct the CS. First, sort the variables in descending order by $\alpha_{lj}$. Then, add variables to the CS until their cumulative probability exceeds ρ. In practice, we may further prune the CSs based on their “purity”, which is defined as the smallest absolute correlation among all pairs of variables within the CS. The reason for doing this is that a CS may lack inferential value because it contains many uncorrelated variables.

**Algorithm 1:** Generalized Iterative Bayesian Stepwise Selection for CoxPH-SuSiE.

**Require:** Data inputs X (n×p matrix), y (p-vector of observed times), and δ (p-vector, 0 = censored, 1 = uncensored).
**Require:** L, the number of single effects.
**Require:** Initial estimates of the prior variances, $\sigma_{0l}^{2}$, l=1,…,L.
**Require:** A function COXPH-SER $\left(X,y,\delta ,c;\sigma_{0}^{2}\right)\to \left(\alpha ,\mu_{1},\sigma_{1}^{2}\right)$ that computes an (approximate) posterior distribution of (b,γ) for an CoxPH-SER model with prior variance $\sigma_{0}^{2}$ given data X, y, δ , c.
Initialize the p-vectors of posterior mean coefficients, ${\bar{b}}_{l}$, l=1,…,L.
Initialize the n-vector of offsets, c=0.
**repeat**
**for** l in 1,…,L **do**
Remove the lth single effect from the offsets, $c_{l}\leftarrow c-X{\bar{b}}_{l}$.
$\left(\alpha_{l},\mu_{l},\sigma_{l}^{2}\right)\leftarrow \text{COXPH-SER}\left(X,y,\delta ,c_{l};\sigma_{0l}^{2}\right)$
Update $\sigma_{0l}^{2}$ using (21), $\sigma_{0l}^{2}\leftarrow \sum_{j=1}^{p}\alpha_{jl}\left(\mu_{jl}^{2}+\sigma_{jl}^{2}\right)$.
Update the posterior mean coefficients, ${\bar{b}}_{l}=\alpha_{l}\circ \mu_{l}$.
Update the offsets, $c\leftarrow c_{l}+X{\bar{b}}_{l}$.
**end**
**until** convergence criterion is met or the maximum number of iterations reached;
**Return:** CoxPH-SuSiE posteriors $\alpha_{1},\ldots ,\alpha_{L},\mu_{1},\ldots ,\mu_{L},\sigma_{1}^{2},\ldots ,\sigma_{L}^{2}$

While the gIBSS algorithm follows the same logic as the IBSS algorithm of Wang et al. (2020), there is an important difference: IBSS can be rigorously justified as optimizing a variational approximation to the posterior distribution of b under the (Gaussian) SuSiE model (Wang et al., 2020), but we are not able to provide a similar result for gIBSS for fitting CoxPH-SuSiE models. As a result, we are not able to prove that gIBSS is optimizing a specific objective function, or that the gIBSS iterations are guaranteed to converge (although we find that they generally do in practice). We therefore view gIBSS as a heuristic generalization of IBSS, and rely on numerical experiments to validate the approximate inferences and demonstrate its good performance in practice.

#### Computational complexity.

The predominant computation in gIBSS is the repeated fitting of single-variable CoxPH regression models to obtain $\hat{b}$, $s^{2}$ (Section 3.1). Within a single gIBSS iteration, these computations are performed once for each of the L single effects and once for each of the p covariates. Since these computations scale linearly with n, the periteration computational complexity of gIBSS is O(npL). Although this is the same as the computational complexity of (Gaussian) SuSiE, unlike Gaussian SuSiE the CoxPH simple single-variable regressions do not admit closed-form solutions, and so CoxPH-SuSiE is considerably slower than Gaussian SuSiE.

Note that fitting the univariate regressions can easily be performed in parallel, so CoxPH-SuSiE can take advantage of multicore architectures to greatly speed up the gIBSS algorithm. We exploited this property when we applied CoxPH-SuSiE to the large UK Biobank data sets in Section 6.

#### Extension for additional covariates.

In a genetic association or fine-mapping analysis, it is often desired to include covariates such as sex and “genetic principal components” to reduce confounding due to population structure (Bycroft et al., 2018; Price et al., 2006). In principle, CoxPH-SuSiE is naturally extended to allow for m additional covariates by including additional terms in the single-variable CoxPH model (4):

(22) $$\begin{array}{rr} {\text{λ}}_{i}(t) & ={\text{λ}}_{0}(t)\text{exp}\left(w^{⊤}z_{i}+bx_{i}+c_{i}\right)\\ b & ∼N\left(0,\sigma_{0}^{2}\right)\\ w & ∼N\left(0,{\text{Σ}}_{w}\right), \end{array}$$

Here, $z_{i}$ denotes the additional covariates for sample i (a vector of length m), the corresponding coefficients are denoted as w, and ${\text{Σ}}_{w}$ denotes the prior covariance of w. However, including these additional covariates in the CoxPH model means that the 1-d integrals in the posterior computations for the single-variable SER would become (m+1)-dimensional integrals. This would greatly increase the computational expense of applying CoxPH-SuSiE to large data sets (such as the UK Biobank data we analyze in Section 6). Therefore, we implemented the following practical approach based on an approximation similar to the approximation used in CoxPH-SuSiE: first we fit a CoxPH regression model that *only* included the additional covariates—that is, (22) with b=0 and $c_{i}=0$, i=1,…,n—then we initialized the offsets $c_{i}$ in the gIBSS algorithm to $c_{i}={\hat{w}}^{⊤}z_{i}$, where $\hat{w}$ was the vector estimated coefficients. This approach imposed only a one-time cost of fitting a CoxPH regression model with m covariates. We used this approach in the CoxPH-SuSiE fine-mapping analyses of asthma, where we included additional covariates for sex and genetic PCs (Section 6).

### 3.4. Alternative approaches.

While developing the approach described above, we also explored alternative approaches to fitting CoxPH-SuSIE models. In one approach, we approximated the Bayes factors using the Asymptotic Bayes Factor (ABF) approximation from Wakefield (2009) instead of the Laplace BF (12). Both approximations use the maximum-likelihood estimate $\hat{b}$ and its variance $s^{2}$, but they use these quantities in different ways. In our experiments (Section 4), we found that the Laplace BF was more accurate than the ABF, so we used the Laplace BF in our CoxPH-SuSiE method.

Another approach we considered built on the “SuSiE-RSS” method from Zou et al. (2022). SuSiE-RSS fits the same Gaussian SuSiE model as Wang et al. (2020), but the computations are different because SuSiE-RSS works with summary statistics instead of individual-level data; specifically, SuSiE-RSS accepts the least-squares estimate of the coefficient, $\hat{b}$, and its variance, $s^{2}$, for each of the p covariates, and the p×p sample correlation matrix. A simple idea then is to provide SuSiE-RSS with the $\hat{b}$, $s^{2}$ obtained from the p CoxPH regression models. This approach is potentially attractive because the single-variable CoxPH regression models need only to be fit once, rather than once per iteration and per single effect in gIBSS. However, in our experiments (see below) this approach performed considerably worse than gIBSS. Note that in the L=1 case, applying SuSiE-RSS in this way corresponds to using the ABF instead of the Laplace BF, and as we noted above the ABF was less accurate than the Laplace BF, providing an additional point against this SuSiE-RSS-based approach.

## 4. Comparison of different approaches to computing the Bayes factors.

In our first set of experiments, we empirically assessed the accuracy of the Laplace Bayes Factor (12) and compared it to the Asymptotic Bayes Factor (13). To provide a “gold standard” to compare to, we computed accurate estimates of the Bayes factors (6) using numerical quadrature methods. [We used Gauss-Hermite quadrature implemented by the gauss.quad.prob function from the statmod R package (Smyth, 2005), with 32 nodes; for details, see the Appendix.] For all the Bayes factors, we fixed the prior variance $\sigma_{0}^{2}$ to 1, and we set the offset to zero.

Since we were ultimately interested in applying these methods to data from UK Biobank with very large sample size (n≈500,000), we simulated genetic data sets with n=500,000 to model this setting. For each simulation, we generated censored TTE data from a “singleSNP” CoxPH model: we a simulated genetic variant—specifically, a single nucleotide polymorphism (SNP)—as $x_{i}∼\text{Binom}(2,f)$, where f was the minor allele frequency (MAF), then we simulated the TTE phenotype from the single-SNP CoxPH model with regression coefficient b=0.1. We simulated data sets with different combinations of censoring rates and MAFs; we simulated 50 data sets for each combination. Additional details on how these censored TTE data were simulated are given in the Appendix.

**FIGURE 1.**
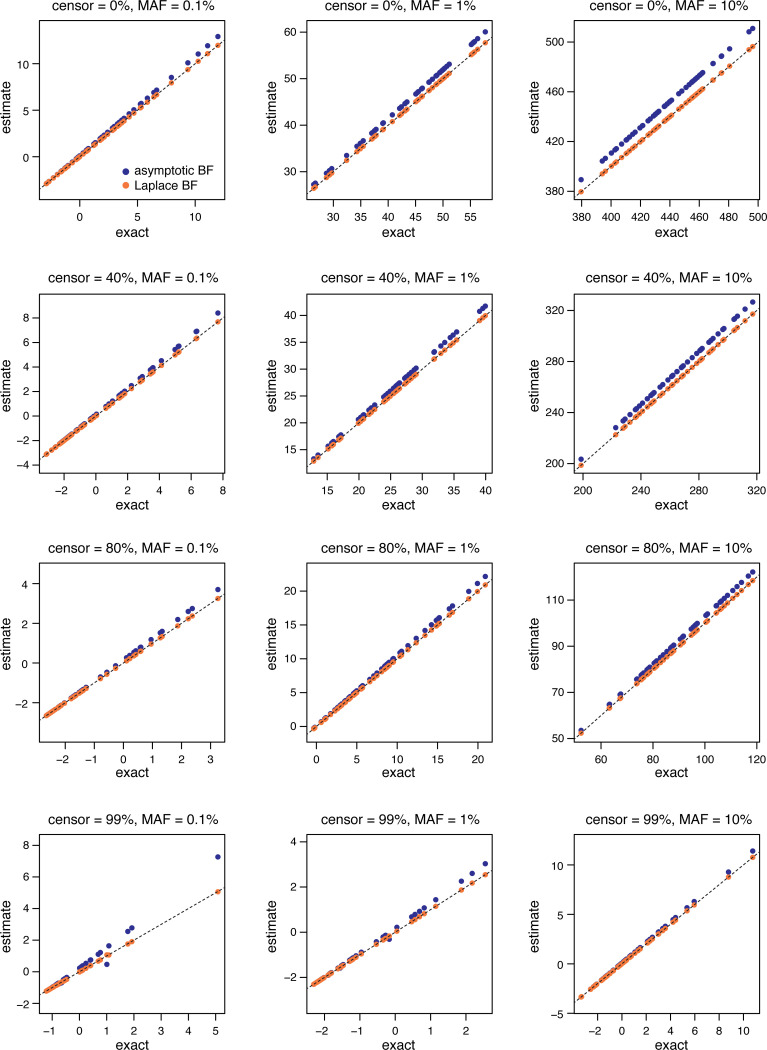
Comparison of (log base-10) Bayes factor estimates in single-SNP simulations with varying censoring rates and SNP minor allele frequencies (MAFs). Each plot shows results from 50 simulations. “Exact” is the numerical quadrature estimate of the Bayes factor (which is not necessarily exact, but should be very accurate).

In these simulations, the Laplace BF was very accurate for all censoring rates and MAFs (Figure 1). Importantly, the Laplace BF achieved good accuracy at a much lower computational cost than the computationally intensive numerical quadrature method. By contrast, the ABF was less accurate in simulations, with a tendency to overestimate the BF. Since the effort involved in computing the Laplace BF and ABF is very similar, the Laplace BF is clearly preferable, and so this is what we used to implement CoxPH-SuSiE.

## 5. Comparison of BVSR methods for fine-mapping TTE phenotypes.

We simulated fine-mapping data sets to assess the performance of CoxPH-SuSiE for the task of fine-mapping TTE phenotypes. In these simulations, we also compared CoxPH-SuSiE against three existing BVSR methods for censored TTE outcomes for which software was available: R2BGLiMS (Newcombe et al., 2017), BVSNLP (Nikooienejad, Wang and Johnson, 2020) and survival.svb (Komodromos et al., 2022). These three methods were implemented as R packages (R Core Team, 2022) and we refer to the methods by the names of their R packages. We also assessed the SuSiE-RSS-based approach which was described in Section 3.4.

To mimic realistic fine-mapping settings, we simulated censored TTE phenotypes using real genotype data from the Genotype-Tissue Expression (GTEx) project (GTEx Consortium et al., 2015; eGTEx Project, 2017) and UK Biobank (Bycroft et al., 2018; Sudlow et al., 2015). The genotype data from GTEx and UK Biobank are both well suited to illustrate fine-mapping due to the high density of available genetic variants. The UK Biobank data in particular are of a much larger sample size and therefore are helpful for assessing the ability of the methods to cope with large-scale data. Both data sets feature genetic variants with complex patterns of correlation and many very strong correlations. Therefore, we did not expect any of the methods to achieve 100% accuracy in identifying the causal variants, even with the larger sample sizes.

We used the GTEx and UK Biobank genotype data to generate two sets of simulations, which are summarized in Table 1. For each of the GTEx simulations, we randomly selected a fine-mapping region containing 1,000 SNPs between base-pair positions 61,597,515 and 63,597,178 on chromosome 1 (Genome Reference Consortium human genome assembly 38). On average, the region containing 1,000 SNPs spanned 280 kb. For each of the UK Biobank simulations, we randomly subsampled 50,000 genotype samples, then we selected a region containing 1,000 SNPs between base-pair positions 18,510,134 and 19,065,757 on chromosome 3 (Genome Reference Consortium human genome assembly 37, hg19). On average, the 1,000-SNP region spanned 300 kb.

For each simulation, we randomly selected a small number of SNPs to be the SNP affecting the TTE outcome, then we simulated censored TTE data following the procedure described in the Appendix. In both sets of simulations, we simulated data sets with different numbers of causal variables (ranging from 1 to 3, as well as the setting in which none of the causal variables affected the phenotype) and different censoring levels. In total, we simulated 400 fine-mapping data sets using the GTEx genotypes and 240 data sets using the UK Biobank data sets. See the Appendix for more details.

Each of the methods has several tuning parameters that may affect the performance and running time of the method; investigating the impact of the tuning parameters is beyond the scope of this experiment, so when possible we followed the guidance given in the publications and in the software documentation. We also adjusted some settings so that the running time of the methods was similar. (Except for SuSiE-RSS, which is much faster than all the other methods for data sets with large sample size because it uses precomputed summary statistics.) For BVSNLP, we called the bvs() function with prep = FALSE to skip the internal data preprocessing step, and we used a Beta-binomial prior for the model space. For R2BGLiMS, we called the R2BGLiMS() function with the Weibull modeling option and a Beta-binomial prior for the model space. For survival.svb, we called the svb.fit() function with the maximum number of iterations set to 1,000 for the GTEx data sets and lowered it to 100 for the UK Biobank data sets to reduce running time. For CoxPH-SuSiE, we used the ibss_from_ser() function from the logisticsusie R package with the number of single effects, L, set to 5 and the prior variance $\sigma_{0}^{2}$ was initialized to 1. (The logisticsusie R package is available at https://github.com/karltayeb/logisticsusie/ and in the Zenodo repository; Yang et al. 2025.) We set the maximum number of iterations to 100 for the GTEx data sets and 10 for the UK Biobank data sets. For SuSiE-RSS, we ran the susie_rss() function from the susieR package (Wang et al., 2020; Zou et al., 2022) with L = 5, var_y = 1 and max_iter = 100. For both CoxPH-SuSiE and SuSiE-RSS, the required summary statistics — that is, the coefficient estimates and their corresponding variances — were computed using the coxph() function from the survival R package (Therneau and Grambsch, 2000).^1^ Other tuning parameters not mentioned were kept at their defaults for all methods. The code used to run these experiments can be found in a git repository on GitHub, https://github.com/yunqiyang0215/survival-susie/ (see also Yang et al. 2025). Other details, including versions of the software used and the computing setup, are given in the Appendix.

As mentioned in the introduction, the two key statistical quantities for fine-mapping are PIPs and CSs. However, since most of the methods do not yield CSs, we focused our comparisons on PIPs. (CoxPH-SuSiE and SuSiE-RSS provide both PIPs and CSs, and we compare the CSs from these two methods below.)

**FIGURE 2.**
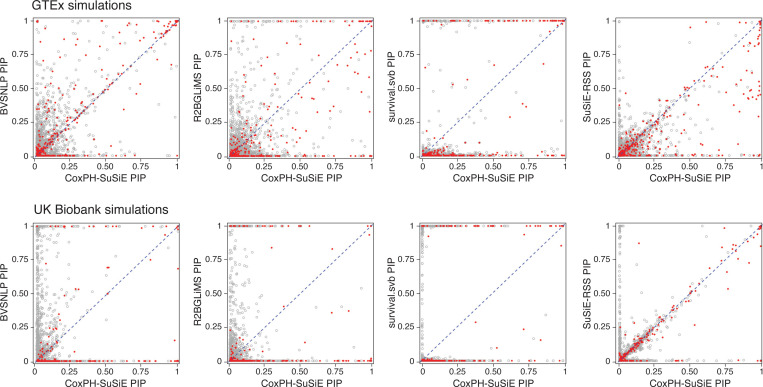
CoxPH-SuSiE PIPs vs. PIPs from other methods. Each point is a variable (SNP); true causal variables (causal SNPs) are shown as solid red circles, and non-causal variables are shown as open gray circles. Each plot in the top row contains 400,000 points (1,000 SNPs × 400 data sets), and each plot in the bottom row contains 240,000 points (1,000 SNPs × 240 data sets). See Supplementary Figures 1 and 2 for a breakdown of these results by censoring level.

**FIGURE 3.**
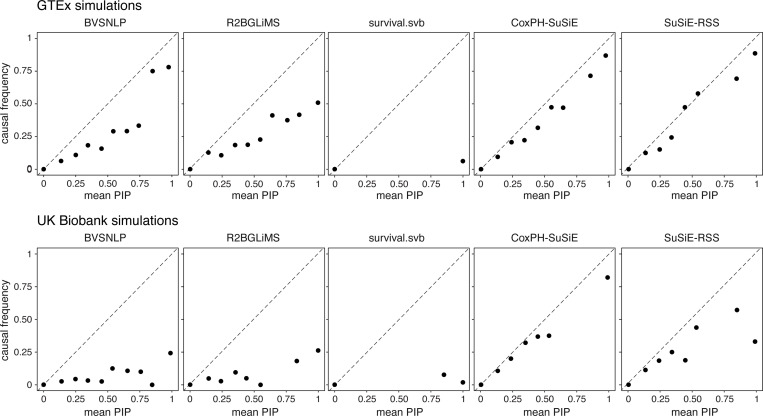
Assessment of PIP calibration. SNPs from all simulations (400 GTEx simulations and 240 UK Biobank simulations) were grouped into bins according to their PIP values (10 equally spaced bins from 0 to 1). The plots then show the average PIP from each bin (*x*-axis) against the proportion of SNPs in that bin that are causal (*y*-axis). Bins with fewer than 10 observations were removed from the plots. A well-calibrated method should produce a plot with points near the diagonal.

First, we found that the different methods, although all taking conceptually similar approaches to the problem, produced strikingly different PIPs (Figure 2). So we examined these differences more closely. One hope is that the PIPs are *well calibrated*; e.g., that among SNPs assigned a PIP near 0.95, approximately 95% should be causal. Among the methods compared, the CoxPH-SuSiE PIPs were best calibrated in both simulation settings, followed by SuSiE-RSS (Figure 3). Correspondingly, most methods overstated the PIPs, particularly in the UK Biobank simulations: among SNPs with PIP 95% or greater, at most 35% actually were causal, except for CoxPH-SuSiE, which identified causal SNPs 84% of the time (which is much better than the other methods, but still leaves some room for improvement). Among all the methods compared, survival.svb was least well calibrated: almost all its PIPs were concentrated near 0 or 1, and the PIPs above 95% were almost always false positives (98% of the time).

**FIGURE 4.**
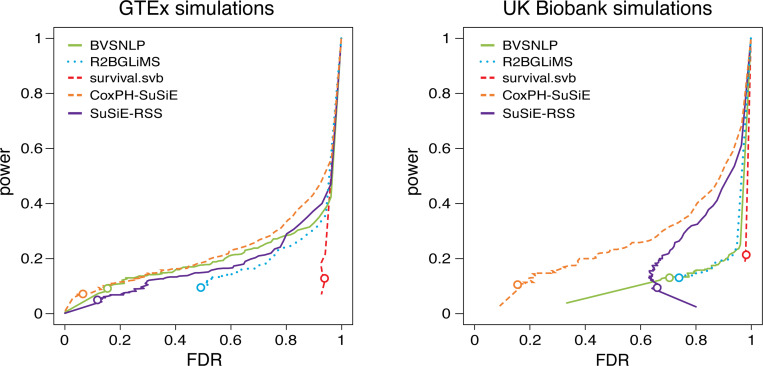
Discovery of causal SNPs using posterior inclusion probabilities (PIPs). Each curve shows power vs. FDR in identifying causal SNPs. FDR and power are defined as FDR:=FP/(TP + FP) and power := TP/(TP+FN), where FP, TP, FN and TN denote the number of false positives, true positives, false negatives and true negatives, respectively. FDR and power were calculated from 400 GTEx simulations and 240 UK Biobank simulations as the PIP threshold was varied from 0 to 1. Note that these plots are the same as precision-recall plots after flipping the *x*-axis because precision = 1 − FDR and recall = power.) Open circles are drawn at a PIP threshold of 0.95. See Supplementary Figures 3 and 4 for the power vs. FDR results stratified by censoring level.

We also compared how effectively the PIPs from different methods identified the causal SNPs by comparing the power and FDR of each method at different PIP thresholds (Figure 4). In the GTEx simulations, CoxPH-SuSiE PIPs almost always had highest power at each FDR threshold, followed closely by BVSNLP and SuSiE-RSS. In the UK Biobank simulations, the CoxPH-SuSiE PIPs far outperformed the PIPs from the other methods. The survival.svb PIPs exhibited particularly poor performance in both simulation settings. As expected, power for all methods went down as censoring increases, but CoxPH-SuSiE maintained the best or among the best performance in all censoring settings (Supplementary Figures 3 and 4).

While CoxPH-SuSiE does require considerable computational effort to run on large data sets, its running times were nonetheless quite comparable to the other methods (Table 2). The one exception was SuSiE-RSS, which uses only summary statistics, and therefore is comparatively fast for data sets with large sample sizes.

**FIGURE 5.**
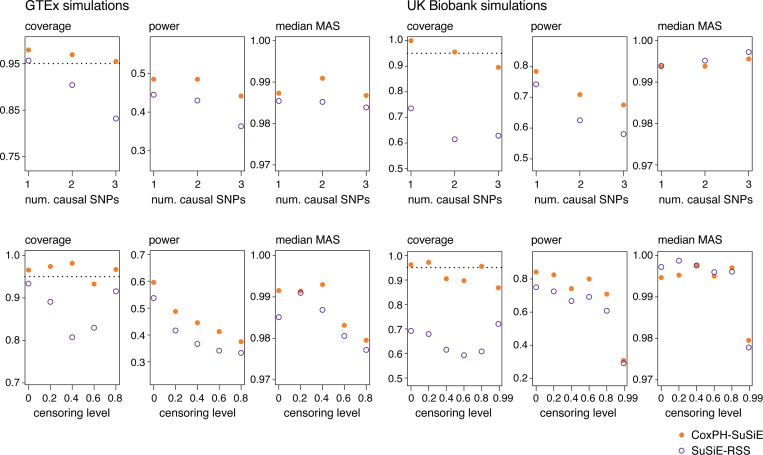
Assessment of 95% credible sets (CSs) from CoxPH-SuSiE and SuSiE-RSS. The performance metrics are: *coverage*, the proportion of CSs that contain a true causal SNP; *power*, the proportion of true causal SNPs included in a CS; and *median MAS*, in which *MAS* is “mean absolute correlation”, the mean of the correlations (Pearson’s r) among all pairs of SNPs within the given CS. The results are stratified by number of causal SNPs in the top row and by censoring level in the bottom row. The target coverage (95%) is shown as a dotted horizontal line. Following Wang et al. (2020); Zou et al. (2022), all CSs returned by CoxPH-SuSiE and SuSiE-RSS with “purity” (the minimum absolute correlation among all pairs of SNPs) less than 0.5 were not considered.

Among the methods compared, only CoxPH-SuSiE and SuSiE-RSS provide CSs. Each CS is meant to capture at least one causal variable (with high probability). A key performance metric is *coverage*, the proportion of CSs that contain at least one true causal SNP. We found that, across the different censoring levels and numbers of causal SNPs, the CoxPH-SuSiE CSs achieved, or came very close to, the target coverage of 95%, whereas the SuSiE-RSS CSs performed less well, with coverage dropping as low as 0.6 (Figure 5). The good coverage of CoxPH-SuSiE CSs did not come at the cost of the other performance metrics: in most scenarios CoxPH-SuSiE outperformed SuSiE RSS in both power and median MAS (Figure 5).

## 6. Application to fine-mapping asthma loci in UK Biobank.

To demonstrate our method on real data, we analyzed age of diagnosis data for asthma in UK Biobank samples (Sudlow et al., 2015). The UK Biobank is a large, population-based prospective study, with detailed phenotype and genotype data from over 500,000 participants in the United Kingdom (ages were between 40 and 69 at time of recruitment). The UK Biobank imputed genotypes feature a high density of available SNPs (Bycroft et al., 2018), so they are well suited for fine-mapping. Several previous studies have performed association analyses for asthma using UK Biobank data (Clay et al., 2022; Ferreira et al., 2019; Han et al., 2020; Pividori et al., 2019; Valette et al., 2021; Vicente, Revez and Ferreira, 2017; Zhu et al., 2018). This includes Bi et al. (2020), who used a CoxPH model to perform their association analysis. But, to our knowledge, only Clay et al. (2022); Zhong et al. (2025); Zhu et al. (2018) took the step of fine-mapping the asthma loci. (Note that Clay et al. 2022 focused on fine-mapping the HLA region.) None of these fine-mapping analyses exploited the available TTE information (that is, the age at which asthma was diagnosed).

**FIGURE 6.**
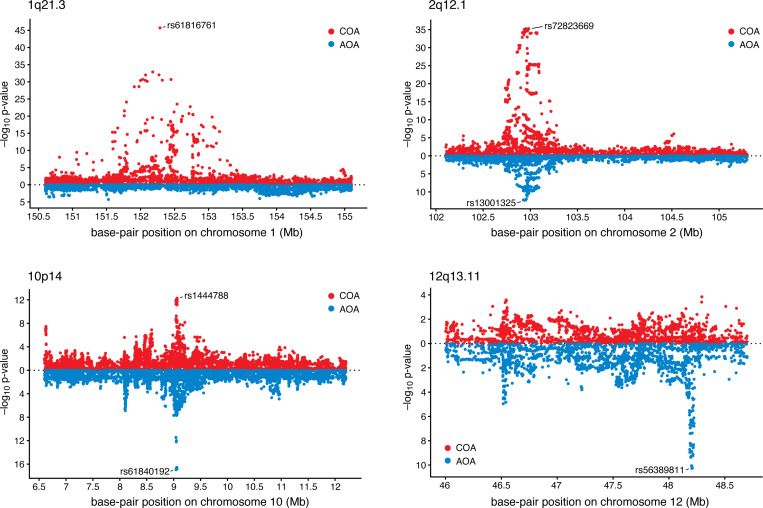
CoxPH-based association analyses of the UK Biobank TTE asthma data for four of the asthma loci identified in Pividori et al. (2019). Each point depicts a SNP. At each locus, the top association for COA and/or AOA is highlighted. The *p*-values were obtained from SPACox (Bi et al., 2020), specifically the p.value.spa column of the SPACox output. Only SNPs with MAF greater than 1% were included in the association analyses. See Table 3 and the Appendix for details on the data used in these association analyses.

Here, we fine-mapped asthma loci by applying CoxPH-SuSiE to the asthma age-of-diagnosis data from UK Biobank. (Specifically, these were self-reported doctor diagnoses; see the Appendix for details on how event times and censoring status were defined.) We selected 8 loci (regions) for fine-mapping from the associations reported in Pividori et al. (2019) (Table 4). Pividori et al. (2019) distinguished between genetic associations for childhood-onset asthma (COA) and adult-onset asthma (AOA), and we selected loci that showed a variety of association patterns: some with associations with both AOA and COA, and others with associations in only one of these two. We performed our own association analyses using a CoxPH model to confirm these patterns of association also hold in a TTE analysis (Figure 6).

If a causal SNP is specific to COA, then including AOA events in the fine-mapping analysis could introduce noise rather than enhance power. Conversely, if a SNP affects both AOA and COA risk, analyzing all asthma events together may be more powerful. We therefore fine-mapped each region using one of three analysis strategies: using childhood onset events for regions showing COA associations only; using adult onset events (ignoring childhood onset cases) for those regions showing AOA associations onlyl and using all asthma (AA) onset events for those regions showing both COA and AOA associations. We used the definitions of COA and AOA from Pividori et al. (2019). At each locus, we included all SNPs with MAFs of 1% or more that were within 250 kb of the top association. We ran CoxPH-SuSiE with L=10 single effects and filtered out CSs with purity less than 0.5. To cope with the large scale of the UK Biobank data, we stopped the gIBSS algorithm after 10 iterations (if the stopping criterion had not already been met). See the Appendix for details on the steps taken to prepare the UK Biobank phenotype and genotype data for the CoxPH-SuSiE analyses. All the code used to generate the results presented here can be accessed at https://github.com/yunqiyang0215/survival-data-analysis. This code is also included in the Zenodo repository (Yang et al., 2025).

**FIGURE 7.**
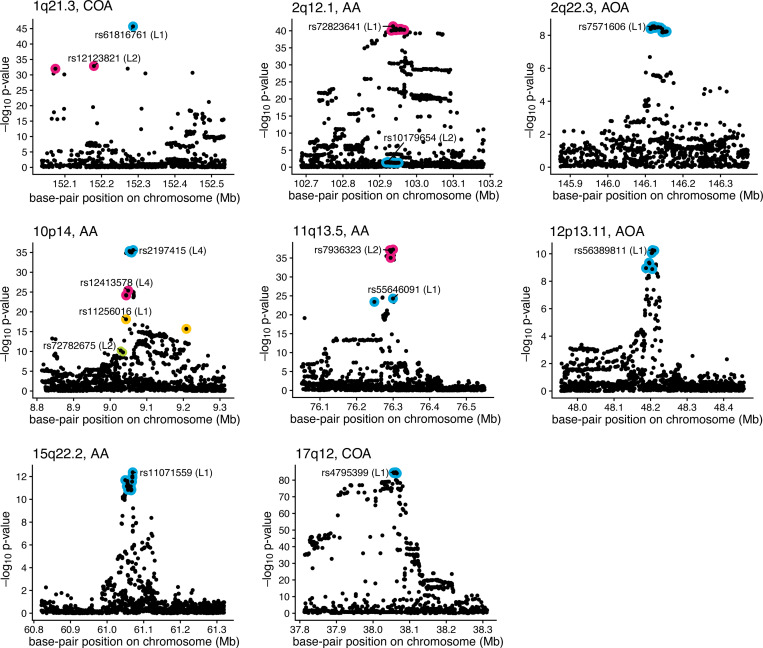
CoxPH-SuSiE asthma fine-mapping results. Each point depicts a single SNP. The *p*-values were computed by SPACox (Bi et al., 2020). Membership of SNPs to CSs is indicated by different colors. The “sentinel SNP” (i.e., the SNP with the highest PIP) in each CS is labeled.

The fine-mapping results are summarized in Figure 7 and Table 5; see the Supplementary Data for more detailed results. At 4 of the 8 asthma loci, CoxPH-SuSiE identified multiple CSs, suggesting the presence of multiple causal variants. At one locus, 10p14, CoxPH-SuSiE identified 4 CSs, suggesting the presence of 4 distinct asthma risk variants. The ability of fine-mapping analyses to identify multiple causal variants underlying an association signal is an important motivation for fine-mapping.

The strongest associations (Table 4) are often expected to show up as SNPs with large PIPs in a CS. Consistent with this, all but one of the top SNPs were also the sentinel SNP in a CS (Table 5), and the other top SNP (rs11236797, at 11q13.5) was one of 10 SNPs included in a CS for that region.

**FIGURE 8.**
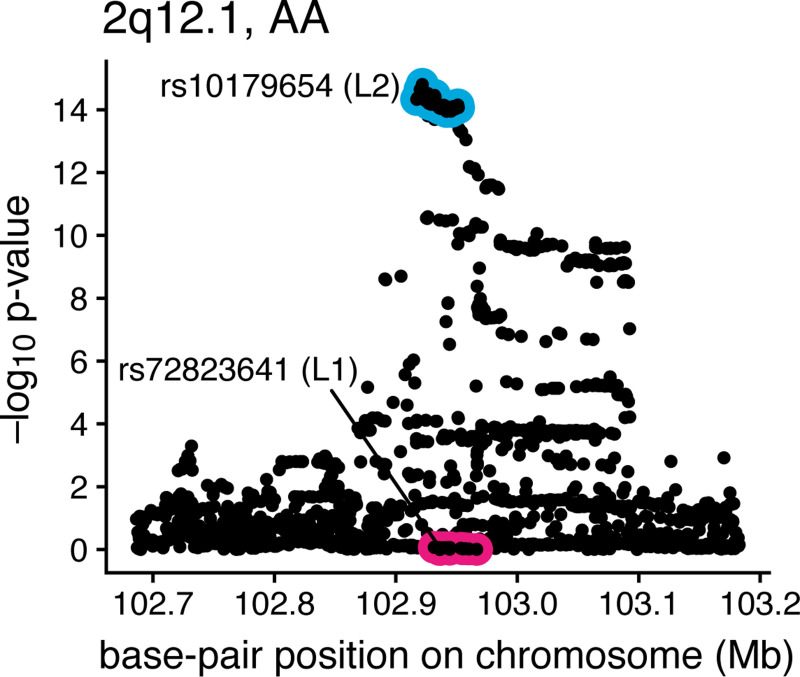
CoxPH-based association analysis of the 2q12.1 asthma locus conditioned on the genotype of a candidate asthma SNP at the same locus. The association tests are conditioned on the genotype of SNP rs72823641, which is the sentinel SNP of the first CS (see Figure 7 and Table 5). The p-values were computed using SPACox, with the genotype of SNP rs72823641 included as an additional covariate. Each point depicts a SNP. The two CSs returned by CoxPH-SuSiE (Figure 7) are shown by the red and blue circles.

The analysis of asthma locus 2q12.1 identified a CS containing SNPs with very little evidence for marginal association (Figure 7). This can happen in a fine-mapping analysis when a signal becomes apparent only after accounting for—i.e., conditioning on—the effects of other causal SNPs. Indeed, the potential to identify such “secondary” signals is another reason to conduct a fine-mapping analysis. To check that this was the explanation here, we performed a “conditional” association analysis for the SNPs at 2q12.1: we conditioned on the genotype of rs72823641 (the top SNP in the first CS) by including it as an additional covariate in the CoxPH model. This association analysis, shown in Figure 8, produced much more significant p-values for the SNPs in the second CS, consistent with the fine-mapping results that suggest the presence of 2 causal SNPs at this locus.

Among the CSs returned by CoxPH-SuSiE, 6 included a SNP with a PIP of 0.5 or greater (Table 5, Supplementary Data). These SNPs are of particular interest because they are likely to be the SNPs that affect asthma risk, or they might tag other genetic variants (unavailable to us) that are the asthma risk variants. For comparison, Zhong et al. (2025), using SuSiE with summary statistics to finemap asthma risk loci, obtained PIP > 0.5 for 3 of these 6 SNPs. They also identified rs12413578 with PIP = 0.44. But two of the causal SNPs we identified the 10p14 locus, rs11256016 and rs72782675, were not included in any of their CSs.

The two SNPs with the highest PIPs were both in the 1q21.3 locus. Both SNPs have been previously reported as pathogenic or tagging pathogenic variants: rs61816761 (PIP > 0.999 for COA and AA) is a stop-gain mutation (c.16819G>A) that occurs in an exon of the gene *FLG* (Palmer et al. 2006; Smith et al. 2006; ClinVar accession VCV000016319.70, OMIM entry 135940); rs12123821 (PIP > 0.93 for COA and AA) tags a loss-of-function mutation in *FLG*, c.2282del4 (Marenholz et al. 2017; OMIM entry 135940). Our fine-mapping results further support these two variants as affecting asthma risk.

The other 4 SNPs with a PIP of 0.5 or greater are all intergenic, non-coding variants for which their regulatory function and pathogenicity are unknown. One SNP, rs55646091 (PIP = 0.61), is not the top association at the 11q13.5 locus, and therefore may have been missed by some previous studies (the top association, rs11236797, is in the other CS at that locus). Interestingly, Tomizuka et al. (2024) also identified rs55646091 as a strong putative causal variant for asthma, although did not attribute a regulatory function to this variant. The three remaining SNPs with PIP > 0.5 are all in the 10p14 locus. Zhong et al. (2025) This locus is interesting because many immune-related traits map to this locus, including rheumatoid arthritis, multiple sclerosis, type 1 diabetes and allergic diseases. Interpreting these genetic associations is challenging because the associated variants are far from any protein coding sequence. However, a very recent study of *GATA3*, which is the closest gene to these putative causal variants, discovered a 44-kb regulatory sequence located approximately 1 Mb downstream of *GATA3* that is a distal enhancer of *GATA3* in Th2 cells (Chen et al., 2023). All three candidate causal SNPs we identified at this locus lie at base-pair positions within this 44-kb regulatory sequence.

For completeness, we ran all three types of fine-mapping analysis (COA, AOA, AA) on each region (Supplementary Data). The results were consistent with expectations from the marginal associations: in regions identified as COA only (e.g., 1q21.3) CSs were identified in the COA fine-mapping analysis but not in AOA analysis; and in regions identified as both COA and AOA (e.g., 10p14) more CSs were sometimes identified in the combined AA analysis than either COA or AOA alone, highlighting the benefits of a combined analysis in these cases.

## 7. Discussion.

In this paper, we presented CoxPH-SuSiE, a new Bayesian variable selection method for censored TTE phenotypes, and illustrated its potential on both simulated and real data.

A core computation in CoxPH-SuSiE is the computation of the Bayes factor for the single-variable CoxPH regression model. This computation is much harder than the corresponding computation for the linear model in SuSiE (where the Bayes factor is available in closed form). The algorithm for fitting CoxPH-SuSiE (Algorithm 1) performs this computation many times; specifically, *pL* times in each iteration of the repeat-until loop, where p is the number of covariates, and L is the number of SERs. To help reduce computation, we proposed an approximate Bayes factor using a variant of Laplace’s method, and in experiments we found that this approximate Bayes factor achieved a good balance of accuracy and speed. This approximation makes CoxPH-SuSiE practical for data sets containing hundreds of thousands of samples and over a thousand covariates.

We also exploited the fact that these Bayes factor computations can easily be performed in parallel to reduce the running times. For example, when we applied CoxPH-SuSiE to the 10p14 asthma locus (n=268,829 individuals, p=1,651 SNPs), running on a machine with a single processor took about 6 hours, whereas running on a multicore machine with 10 processors reduced the running time to just over 1 hour. (Details on the computing environment used to obtain these running times are given in the Appendix.) There is potential to further speed up CoxPH-SuSiE by exploiting fast implementations of the CoxPH model in R packages such as SPACox (Bi et al., 2020), COXMEG (He and Kulminski, 2020) or Colossus (Giunta et al., 2024) (See Allignol and Latouche 2025 for a survey of R packages implementing the CoxPH model.) We used the survival R package (Therneau and Grambsch, 2000) in our implementation because it is a robust and widely used software package, but potentially any CoxPH software implementation could be used provided that it gives the three statistics needed to compute the Laplace Bayes factor (eq. 12): the maximum-likelihood estimate of b, its variance, and the likelihood ratio.

There are also practical issues that one should consider when using CoxPH-SuSiE. First, it is important to consider whether the “proportional hazards” assumption is reasonable. In our asthma fine-mapping analyses, we divided asthma cases into childhood-onset asthma and adult-onset asthma using the age thresholds from Pividori et al. (2019). However, these thresholds are somewhat arbitrary, and a more systematic choice of the age thresholds could itself be an interesting problem in the study of asthma subtypes. For TTE data where the proportional hazards assumption does not hold, a better approach might be to incorporate time-varying effects into the CoxPH model (Ojavee et al., 2023). Other TTE models that have been used in genome-wide association studies include frailty models (Dey et al., 2022) and age-dependent liability models (Pedersen et al., 2023). Potentially, these models could also be combined with the SuSiE prior to create alternative fine-mapping methods for TTE phenotypes.

Second, we introduced a model-fitting procedure which we called “Generalized Iterative Bayesian Stepwise Selection”, or “gIBSS” for short (Algorithm 1). This algorithm generalizes the IBSS algorithm for SuSiE (Wang et al., 2020). However, unlike IBSS, there is no guarantee that gIBSS will converge to a stationary point of a specific objective function. Deriving a model-fitting procedure that can be understood as optimizing an approximate posterior—that is, a variational inference method (Blei, Kucukelbir and McAuliffe, 2017)—and understanding the exact form of the approximate posterior are open research questions.

## Supplementary Material

Supplement 1

Table in CSV format giving the results of the CoxPH-SuSiE asthma fine-mapping analyses.

## Figures and Tables

**TABLE 1:** Summary of the settings for the fine-mapping simulations.

genotypes	sample size	covariates (SNPs)	effect variance	average region size

GTEx	574	1,000	1	280 kb
UK Biobank	50,000	1,000	0.1	300 kb

**TABLE 2:** Running times in seconds of the different methods in the fine-mapping simulations. The first number is the average across all simulations; the numbers in parentheses give the full range across the simulations.

method	GTEx	UK Biobank

BVSNLP	58 (15.7–2,042)	4,499 (490–79,601)
R2BGLiMS	190 (167–494)	10,463 (8,870–14,118)
survival.svb	139 (6–844)	2,496 (118–15,353)
CoxPH-SuSiE	331 (80–1,014)	7,275 (4,472–14,416)
SuSiE-RSS	6.4 (6.0–7.6)	218 (199–296)

**TABLE 3:** Summary of the TTE data used in the asthma association analyses and asthma fine-mapping analyses.

trait	observed	censored

COA	8,024	260,805
AOA	18,569	242,399
AA	31,860	241,683

**TABLE 4:** Asthma loci from Pividori et al. (2019) fine-mapped using CoxPH-SuSiE. The “traits” column gives the disease phenotypes (COA and/or AOA) that have significant associations in the region. The “top SNP” is the SNP with the strongest association (smallest SPACox association test p-value) in that region for either trait. The chromosomal base-pair positions are based on human genome assembly 19 (Genome Reference Consortium Human Build 37, February 2009)

locus	traits	top SNP	region (bp)	size (kb)	SNPs

1q21.3	COA	rs61816761	152,037,453–152,535,675	498.22	1,333
2q12.1	COA, AOA	rs72823641	102,686,430–103,182,687	496.26	1,821
2q22.3	AOA	rs7571606	145,873,121–146,372,665	499.54	1,190
10p14	COA, AOA	rs2197415	8,813,629–9,312,765	499.14	1,651
11q13.5	COA, AOA	rs11236797	76,050,271–76,549,513	499.24	1,628
12q13.11	AOA*	rs56389811	47,955,425–48,455,255	499.83	1,595
15q22.2	COA, AOA	rs11071559	60,820,809–61,319,821	499.01	1,558
17q12	COA	rs4795399	37,812,435–38,311,433	499.00	1,212

* The 12q13.11 locus was classified as both COA and AOA in Pividori et al. (2019) based on additional analyses, but showed significant association only in AOA so we treated it as AOA only.

**TABLE 5:** Summary of the CoxPH-SuSiE asthma fine-mapping results. The columns in the table from left to right are: asthma locus; trait analyzed (COA = childhood-onset asthma; AOA = adult-onset asthma; AA = all asthma); the credible set (CS) label used in Figure 7; number of SNPs in the 95% CS; *purity*, defined as the smallest absolute correlation (Pearson’s r) among all SNP pairs in the CS (Wang et al., 2020); the sentinel SNP (the SNP with the largest PIP in the CS); the SPACox association test p-value for the sentinel PIP; the PIP of the sentinel SNP; and “candidate genes”, a non-comprehensive listing of candidate asthma genes based on the fine-mapping results and previous asthma GWAS. More comprehensive and detailed results are provided in the Supplementary Data.

region	trait	CS	size	purity	sentinel SNP	p-value	PIP	alleles*	candidate gene(s)

1q21.3	COA	L1	1	–	rs61816761	1.95×10^−46^	>0.99	G/A	*FLG*, *FLG2*, *HRNR*
		L2	2	0.850	rs12123821	1.35×10^−33^	0.93	C/T	*FLG*, *HRNR*, *RPTN*
2q12.1	AA	L1	18	0.986	rs72823641	4.73×10^−42^	0.47	A/T	*IL1R1*, *IL18R1*, *IL1RL2*
		L2	61	0.992	rs10179654	3.01×10^−02^	0.06	G/T	*IL1R1*, *IL18R1*, *IL1RL2*
2q22.3	AOA	L1	32	0.933	rs7571606	2.84×10^−09^	0.05	A/T	*TEX41*, *ACVR2A*
10p14	AA	L1	2	0.887	rs11256016	7.63×10^−19^	0.84	G/A	*GATA3*
		L2	2	0.970	rs72782675	6.10×10^−11^	0.77	T/C	*GATA3*
		L3	3	0.980	rs12413578	4.27×10^−26^	0.64	T/C	*GATA3*
		L4	10	0.996	rs2197415	2.47×10^−36^	0.23	G/T	*GATA3*
11q13.5	AA	L1	2	0.943	rs55646091	5.11×10^−25^	0.61	G/A	*EMSY*, *LRRC32*, *THAP12*
		L2	10	0.900	rs7936323	1.07×10^−37^	0.18	G/A	*EMSY*, *LRRC32*, *THAP12*
12q13.11	AOA	L1	9	0.909	rs56389811	5.36×10^−11^	0.22	T/C	*HDAC7*, *SLC48A1*
15q22.2	AA	L1	18	0.770	rs11071559	4.23×10^−13^	0.25	T/C	*RORA*, *ANXA2*
17q12	COA	L1	8	0.999	rs4795399	1.97×10^−85^	0.19	C/T	*GSDMB*, *ORMDL3*

* Alleles are reported as A/B, where B is the allele that increases the hazard function.

## Footnotes

1 The likelihood ratio $\ell (\hat{b};x,c)/\ell (0;x,c)$ is also needed to compute the Laplace BFs (12) in CoxPH-SuSiE. The survival package provides this quantity for the CoxPH model.

## APPENDIX A: DERIVATIONS

This section contains derivations supporting results in the main text.

### A.1. EM for estimating the prior variance in the SER CoxPH model.

Here we derive the EM update (21) for estimating the prior variance in the SER CoxPH model (defined in Section 3.2 of the main text).

To derive the EM update, we use the multinomial random variable γ for the data augmentation. With this data augmentation, the complete data log-likelihood is

(23)
$$𝓘\left(\sigma_{0}^{2}\right)=\sum_{j=1}^{p}\delta_{1}\left(\gamma_{j}\right)\;\text{log}\;p\left(b_{j}|\sigma_{0}^{2}\right),$$

where $\delta_{y}(x)$ denotes the Dirac “delta” function that is 1 at x=y, and zero at any x≠0. where “constant” absorbs the additional terms in the log-likelihood that do not depend on $\sigma_{0}^{2}$.

The M-step is derived by solving for the $\sigma_{0}^{2}$ that maximizes the expected complete loglikelihood. As with the other calculations for this model, we maximize an approximate expected completed log-likelihood,

(24)
$$E\left[𝓘\left(\sigma_{0}^{2}\right)\right]=-\frac{1}{2}\text{log}\;\sigma_{0}^{2}-\frac{1}{2\sigma_{0}^{2}}\sum_{j=1}^{p}E\left[b_{j}^{2}|\gamma_{j}=1\right]p\left(\gamma_{j}=1\right)+\text{constant}\;\;\approx -\frac{1}{2}\text{log}\;\sigma_{0}^{2}-\frac{\sum_{j=1}^{p}\alpha_{j}\left(\mu_{1j}^{2}+\sigma_{1j}^{2}\right)}{2\sigma_{0}^{2}}+\text{constant}.$$

Taking the derivative of this approximate expected complete log-likelihood, setting it to zero, and solving for $\sigma_{0}^{2}$ results in the closed-form solution (21).

### A.2. Exponential distribution property.

Let X and Y be exponentially distributed with rates λ and μ, respectively. Then $\text{Pr}(X>Y)=\frac{\mu }{\mu +\text{λ}}$.

Proof.

(25)
$$\begin{array}{ll} \text{Pr}(X>Y) & =\int_{0}^{\infty }\int_{0}^{x}\text{λ}e^{-\text{λ}x}\mu e^{-\mu y}dy\;dx\\ & =\int_{0}^{\infty }\text{λ}e^{-\text{λ}x}dx-\int_{0}^{\infty }\text{λ}e^{-\text{λ}x-\mu x}dx\\ & =1-\frac{\text{λ}}{\text{λ}+\mu }\int_{0}^{\infty }(\text{λ}+\mu )e^{-(\text{λ}+\mu )x}dx\\ & =\frac{\mu }{\text{λ}+\mu }. \end{array}$$

## APPENDIX B: COMPUTING THE BAYES FACTORS USING GAUSSIAN-HERMITE QUADRATURE

Here we briefly describe the numerical integration technique, Gauss-Hermite quadrature, that was used to accurately estimate the Bayes factors (12) for the simple single-variable CoxPH regression model (4).

Naylor and Smith (1982) give the following approximation formula (eq. 9 in their paper):

(26)
$$\int_{-\infty }^{\infty }f(t)\phi \left(t;\mu ,\sigma^{2}\right)dt\approx \frac{1}{\sqrt{\pi }}\sum_{i=1}^{n}w_{i}f\left(t_{i}\right),$$

where $\phi \left(x;\mu ,\sigma^{2}\right)$ denotes the probability density function of the normal distribution with mean μ and variance $\sigma^{2}$ at x, and $t_{i}:=\mu +\sqrt{2}\sigma r_{i}$, in which the $r_{i}$ are the zeros of the nth-order Hermite polynomial, with corresponding weights $w_{i}$. See also Davis and Rabinowitz (1985). This formula can be used to approximate an integral $I=\int_{-\infty }^{\infty }g(t)dt$ by expressing it as

(27)
$$I=\int_{-\infty }^{\infty }g(t)dt=\int_{-\infty }^{\infty }h(t)\phi \left(t;\mu ,\sigma^{2}\right)dt$$

where $h(t):=g(t)/\phi \left(t;\mu ,\sigma^{2}\right)$. Here, μ and $\sigma^{2}$ are arbitrary, and so should be chosen to make the approximation as accurate as possible. Results in Liu and Pierce (1994) suggest that μ, $\sigma^{2}$should be chosen so that $g(x)\approx \alpha \phi \left(x;\mu ,\sigma^{2}\right)$ for some constant α.

The numerator in the BF (6) is an integral, I, with $g(t)=\ell (t;x,c)\phi \left(t;0,\sigma_{0}^{2}\right)$, which is proportional to the posterior distribution of b (5). We therefore select μ and $\sigma^{2}$ to be the approximate posterior mean (11) and approximate posterior variance (10), then apply the approximation formula (26) with n=32.

Note that the special case of (26) with n=1 recovers the Laplace BF (12). Larger values of n will provide increasingly accurate approximations of the BF. See Liu and Pierce (1994) for more discussion on the relationship between Laplace’s method and Gauss-Hermite quadrature.

## APPENDIX C: PREPARATION OF THE DATA FOR THE SIMULATIONS AND ASTHMA FINE-MAPPING ANALYSES

### C.1. GTEx data for simulations.

We used genotype data from release v7 of the GTEx Project (GTEx Consortium et al., 2015; eGTEx Project, 2017) to simulate fine-mapping data sets. We arbitrarily chose a region on chromosome 1 near gene *ANGPTL3* as the candidate fine-mapping region. After removing SNPs with minor allele frequencies (MAFs) less than 1%, the GTEx genotype data consisted of genotypes from 574 individuals at 7,154 SNPs.

### C.2. UK Biobank data for simulations.

We used version 3 of the imputed UK Biobank genotypes (Bycroft et al., 2018) to simulate fine-mapping data sets. We arbitrarily chose a region on chromosome 3 near gene *SATB1-AS1* as the candidate fine-mapping region. After removing SNPs with MAFs less than 0.1%, the UK Biobank genotype data consisted of genotypes from 248,980 individuals at 3,119 SNPs. The exact steps taken to prepare these genotype data, including steps to remove individuals based on certain criteria, are described in Zou et al. (2023).

For the simulations, SNPs with MAFs less than 1% were also removed, then further subsampled, separately for each simulation, to obtain 1,000 SNPs. Also, the individuals were subsampled at random, separately for each simulation, to obtain a random set of 50,000 individuals.

### C.3. UK Biobank data for asthma association and fine-mapping analyses.

We used a subset of available UK Biobank genotypes (version 3), removing samples that met one or more of the following criteria for exclusion (similar to the exclusion criteria used in Zou et al. 2023): mismatch between self-reported and genetic sex; withdrew from UK Biobank; didn’t know, or didn’t wish to answer, their asthma diagnosis age. Additionally, we excluded outlying genotype samples based on heterozygosity and/or rate of missing genotypes as defined by UK Biobank (data field 22027), and we removed any individuals having at least one relative in the cohort based on UK Biobank kinship calculations (samples with a value other than zero in data field 22021). Finally, to limit confounding due to population structure, we included only genotype samples marked as “White British” (stored in data field 22009, and based on a PCA of the genotypes; Bycroft et al. 2018). After filtering genotype samples according to these criteria, 273,543 samples remained.

Censored/uncensored status for the three asthma phenotypes was defined as follows. For AA, an individual was uncensored if a doctor diagnosis for asthma was reported (data fields 3786, 22147); a censored individual was any other individual. Using the same data fields, COA uncensored individuals were individuals who developed asthma at or before age 12, and censored were individuals who developed asthma at or after age 26, or did not report an asthma diagnosis. AOA uncensored individuals were defined as individuals who developed asthma between (and including) ages 26 and 65, and controls were individuals who did not report an asthma diagnosis during the course of the study. These phenotype definitions come from Pividori et al. (2019). For uncensored individuals, the event time was defined as the age of diagnosis (in years) as described above. For censored individuals, the censoring times were defined as follows: for AA, the age at the most recent visit to the assessment center (data field 21003); for COA, always 12 years; for AOA, 65 years or current age, whichever was smaller. Table 3 in the main text summarizes the TTE data for the three asthma phenotypes.

The following covariates were included in the SPACox association analyses and the CoxPH-SuSiE fine-mapping analyses: sex (UK Biobank data field 31) and genetic PCs 1– 10 (data field 22009).

## APPENDIX D: DETAILS OF THE SIMULATIONS

Here we describe the steps that were taken to simulate the fine-mapping data sets.

Simulating censored TTE data requires three inputs: an n×p design matrix, X; $p_{1}\leq p$, the number of effect variables; the mean μ and variance $\sigma^{2}$ for simulating the coefficients of the effect variables; and the censoring level, r∈[0,1). In the comparisons of the different BVSR methods, X was always a genotype data matrix from a real data set. In the Bayes factor comparisons, X was an n×1 matrix containing the genotypes for a single simulated SNP, $x_{i}∼\text{Binom}(2,f)$, where f∈(0,0.5] is the SNP minor allele frequency.

The following simulation procedure generates a vector of event times, $y={\left(y_{1},\ldots ,y_{n}\right)}^{⊤}$, and a vector giving the censoring status for each sample, $\delta ={\left(\delta_{1},\ldots ,\delta_{n}\right)}^{⊤}$:

1. Randomly sample without replacement the $p_{1}\leq p$ indices of the effect variables from {1,…,p}.
2. Simulate the vector of coefficients, $b={\left(b_{1},\ldots ,b_{p}\right)}^{⊤}$: if j∈{1,…,p} is one of the indices selected in the first step, $b_{j}∼N\left(\mu ,\sigma^{2}\right)$; otherwise, $b_{j}=0$. Note that in the Bayes factor comparisons, we set $\sigma^{2}=0$ so that all $b_{j}$ were either 0 or μ, with μ=0.1.
3. For each sample i=1,…,n, compute the survival rate as $\lambda_{i}^{s}=b_{0}+x_{i}^{⊤}b$. In all our simulations, we set $b_{0}=1$.
4. For each sample i=1,…,n, simulate the survival time and censor time as $T_{i}∼$ Expon $\left({\text{λ}}_{i}^{s}\right)$ and $C_{i}∼$ Expon $\left({\text{λ}}^{c}\right)$, where ${\text{λ}}^{c}=\frac{r{\bar{\text{λ}}}^{s}}{1-r}$, and ${\bar{\text{λ}}}^{s}=\frac{1}{n}\sum_{i=1}^{n}{\text{λ}}_{i}^{s}$. See below for discussion on the formula for ${\text{λ}}^{c}$.
5. Finally, record the event time and censoring status: $y_{i}=\text{min}\left(T_{i},C_{i}\right)$ and $\delta_{i}$ is 1 if $T_{i}\leq C_{i}$, otherwise it is zero.

Note that data simulated in this way satisfies the proportional hazard assumption.

Regarding the censoring rate, ${\text{λ}}^{c}$, we would ideally like ${\text{λ}}^{c}$ to satisfy

(28)
$$r=\frac{1}{n}E\left[\sum_{i}^{n}\text{I}\left(C_{i}<T_{i}\right)\right]=\frac{1}{n}\sum_{i=1}^{n}\text{Pr}\left(C_{i}<T_{i}\right)=\frac{1}{n}\sum_{i=1}^{n}\frac{{\text{λ}}^{c}}{{\text{λ}}_{i}^{s}+{\text{λ}}^{c}},$$

in which the last equality uses the result from Section A.2. Therefore, the choice ${\text{λ}}^{c}=\frac{r{\bar{\text{λ}}}^{s}}{1-r}$ is an approximation to this ideal choice intended to simplify the simulation procedure.

## APPENDIX E: COMPUTING ENVIRONMENT

The simulations were performed using R 4.2.0 (R Core Team, 2022), linked to the OpenBLAS 0.3.13 optimized numerical libraries, on Linux machines (Scientific Linux 7.4) with Intel Xeon E5–2680v4 (“Broadwell”) processors. The following R packages were used: BVSNLP 1.1.9, R2BGLiMS 0.1–07-02–2020, survival 3.3–1, susieR 0.12.35, survival.svb 0.0–2 and statmod 1.5.0. All computations in the simulations were performed using a single processor.

The asthma fine-mapping analyses were performed using R 4.3.1, linked to the OpenBLAS 0.3.20 optimized numerical libraries, on Linux Machines with Intel Xeon Gold 6326 processors. The following R packages were used: susieR X, survival X and SPACox X. Also, PLINK v2.00a2LM 64-bit Intel (21 Feb 2019) (Chang et al., 2015) was used to prepare the UK Biobank genotype data (Section C).

## References

[R1] ALLIGNOL A. and LATOUCHE A. (2025). CRAN task view: survival analysis. Version 2025–02-09.

[R2] BENNER C., SPENCER C. C. A., HAVULINNA A. S., SALOMAA V., RIPATTI S. and PIRINEN M. (2016). FINEMAP: efficient variable selection using summary data from genome-wide association studies. Bioinformatics 32 1493–1501. 26773131 10.1093/bioinformatics/btw018 PMC4866522

[R3] BI W., FRITSCHE L. G., MUKHERJEE B., KIM S. and LEE S. (2020). A fast and accurate method for genome-wide time-to-event data analysis and its application to UK Biobank. American Journal of Human Genetics 107 222–233. 32589924 10.1016/j.ajhg.2020.06.003 PMC7413891

[R4] BLEI D. M., KUCUKELBIR A. and MCAULIFFE J. D. (2017). Variational inference: a review for statisticians. Journal of the American statistical Association 112 859–877.

[R5] BYCROFT C., FREEMAN C., PETKOVA D., BAND G., ELLIOTT L. T., SHARP K., MOTYER A., VUKCEVIC D., DELANEAU O., O’CONNELL J. (2018). The UK Biobank resource with deep phenotyping and genomic data. Nature 562 203–209. 30305743 10.1038/s41586-018-0579-z PMC6786975

[R6] CALLAS P. W., PASTIDES H. and HOSMER D. W. (1998). Empirical comparisons of proportional hazards, Poisson, and logistic regression modeling of occupational cohort data. American Journal of Industrial Medicine 33 33–47. 9408527 10.1002/(sici)1097-0274(199801)33:1<33::aid-ajim5>3.0.co;2-x

[R7] CHANG C. C., CHOW C. C., TELLIER L. C., VATTIKUTI S., PURCELL S. M. and LEE J. J. (2015). Second-generation PLINK: rising to the challenge of larger and richer datasets. Gigascience 4 s13742–015–0047–8. 10.1186/s13742-015-0047-8 PMC4342193 25722852

[R8] CHEN W., LARRABEE B. R., OVSYANNIKOVA I. G., KENNEDY R. B., HARALAMBIEVA I. H., POLAND G. A. and SCHAID D. J. (2015). Fine mapping causal variants with an approximate Bayesian method using marginal test statistics. Genetics 200 719–736. 25948564 10.1534/genetics.115.176107 PMC4512539

[R9] CHEN H. V., LORENZINI M. H., LAVALLE S. N., SAJEEV K., FONSECA A., FIAUX P. C., SEN A., LUTHRA I., HO A. J., CHEN A. R., GURUVAYURAPPAN K., O’CONNOR C. and MCVICKER G. (2023). Deletion mapping of regulatory elements for GATA3 in T cells reveals a distal enhancer involved in allergic diseases. American Journal of Human Genetics 110 703–714. 36990085 10.1016/j.ajhg.2023.03.008 PMC10119147

[R10] CLAY S. M., SCHOETTLER N., GOLDSTEIN A. M., CARBONETTO P., DAPAS M., ALTMAN M. C., ROSASCO M. G., GERN J. E., JACKSON D. J., IM H. K., STEPHENS M., NICOLAE D. L. and OBER C. (2022). Fine-mapping studies distinguish genetic risks for childhood- and adult-onset asthma in the HLA region. Genome Medicine 14 55. 35606880 10.1186/s13073-022-01058-2 PMC9128203

[R11] GTEX CONSORTIUM, ARDLIE K. G., DELUCA D. S., SEGRÈ A. V., SULLIVAN T. J., YOUNG T. R., GELFAND E. T., TROWBRIDGE C. A., MALLER J. B., TUKIAINEN T. (2015). The Genotype-Tissue Expression (GTEx) pilot analysis: multitissue gene regulation in humans. Science 348 648–660. 25954001 10.1126/science.1262110 PMC4547484

[R12] COX D. R. (1972). Regression models and life-tables. Journal of the Royal Statistical Society, Series B 34 187–202.

[R13] COX D. R. (1975). Partial likelihood. Biometrika 62 269–276.

[R14] DAVIS P. J. and RABINOWITZ P. (1985). Methods of numerical integration, 2nd ed. Academic Press, Orlando, FL.

[R15] DEMPSTER A. P., LAIRD N. M. and RUBIN D. B. (1977). Maximum likelihood estimation from incomplete data via the EM algorithm. Journal of the Royal Statistical Society, Series B 39 1–38.

[R16] DEY R., ZHOU W., KIISKINEN T., HAVULINNA A., ELLIOTT A., KARJALAINEN J., KURKI M., QIN A., FINNGEN LEE, et al S.. (2022). Efficient and accurate frailty model approach for genome-wide survival association analysis in large-scale biobanks. Nature Communications 13 5437. 10.1038/s41467-022-32885-x PMC9481565 36114182

[R17] EGTEX PROJECT (2017). Enhancing GTEx by bridging the gaps between genotype, gene expression, and disease. Nature Genetics 49 1664–1670. 29019975 10.1038/ng.3969 PMC6636856

[R18] FERREIRA M. A., MATHUR R., VONK J. M., SZWAJDA A., BRUMPTON B., GRANELL R., BREW B. K., ULLEMAR V., LU Y., JIANG Y. (2019). Genetic architectures of childhood-and adult-onset asthma are partly distinct. American Journal of Human Genetics 104 665–684. 30929738 10.1016/j.ajhg.2019.02.022 PMC6451732

[R19] GEORGE E. I. and MCCULLOCH R. E. (1997). Approaches to Bayesian variable selection. Statistica Sinica 7 339–373.

[R20] GIUNTA E., BAHADORI A., ANDRESEN D., WALSH L., FRENCH B., DAUER L. and BOICE JR J. (2024). Colossus: risk model regression and analysis with complex non-linear models.

[R21] GREEN M. S. and SYMONS M. J. (1983). A comparison of the logistic risk function and the proportional hazards model in prospective epidemiologic studies. Journal of Chronic Diseases 36 715–723. 6630407 10.1016/0021-9681(83)90165-0

[R22] HAN Y., JIA Q., JAHANI P. S., HURRELL B. P., PAN C., HUANG P., GUKASYAN J., WOODWARD N. C., ESKIN E., GILLILAND F. D., AKBARI O., HARTIALA J. A. and ALLAYEE H. (2020). Genome-wide analysis highlights contribution of immune system pathways to the genetic architecture of asthma. Nature Communications 11 1776. 10.1038/s41467-020-15649-3 PMC7160128 32296059

[R23] HE L. and KULMINSKI A. M. (2020). Fast algorithms for conducting large-scale GWAS of age-at-onset traits using Cox mixed-effects models. Genetics 215 41–58. 32132097 10.1534/genetics.119.302940 PMC7198273

[R24] HORMOZDIARI F., KOSTEM E., KANG E. Y., PASANIUC B. and ESKIN E. (2014). Identifying causal variants at loci with multiple signals of association. Genetics 198 497–508. 25104515 10.1534/genetics.114.167908 PMC4196608

[R25] HUGHEY J. J., RHOADES S. D., FU D. Y., BASTARACHE L., DENNY J. C. and CHEN Q. (2019). Cox regression increases power to detect genotype-phenotype associations in genomic studies using the electronic health record. BMC Genomics 20 805. 31684865 10.1186/s12864-019-6192-1 PMC6829851

[R26] HUTCHINSON A., ASIMIT J. and WALLACE C. (2020). Fine-mapping genetic associations. Human Molecular Genetics 29 R81–R88. 32744321 10.1093/hmg/ddaa148 PMC7733401

[R27] IBRAHIM J. G., CHEN M.-H. and SINHA D. (2001). Bayesian survival analysis. Springer, New York, NY.

[R28] KALBFLEISCH J. D. (1978). Non-parametric Bayesian analysis of survival time data. Journal of the Royal Statistical Society, Series B 40 214–221.

[R29] KASS R. E. and RAFTERY A. E. (1995). Bayes Factors. Journal of the American Statistical Association 90 773–795.

[R30] KICHAEV G., YANG W.-Y., LINDSTROM S., HORMOZDIARI F., ESKIN E., PRICE A. L., KRAFT P. and PASANIUC B. (2014). Integrating functional data to prioritize causal variants in statistical fine-mapping studies. PLoS Genetics 10 e1004722. 25357204 10.1371/journal.pgen.1004722 PMC4214605

[R31] KOMODROMOS M., ABOAGYE E. O., EVANGELOU M., FILIPPI S. and RAY K. (2022). Variational Bayes for high-dimensional proportional hazards models with applications within gene expression. Bioinformatics 38 3918–3926. 35751586 10.1093/bioinformatics/btac416 PMC9364383

[R32] KOTE-JARAI Z., SAUNDERS E. J., LEONGAMORNLERT D. A., TYMRAKIEWICZ M., DADAEV T., JUGURNAUTH-LITTLE S., ROSS-ADAMS H., AL OLAMA A. A., BENLLOCH S., HALIM S. (2013). Fine-mapping identifies multiple prostate cancer risk loci at 5p15, one of which associates with TERT expression. Human Molecular Genetics 22 2520–2528. 23535824 10.1093/hmg/ddt086 PMC3658165

[R33] LEE Y., FRANCESCA L., PIQUE-REGI R. and WEN X. (2018). Bayesian multi-SNP genetic association analysis: control of FDR and use of summary statistics. bioRxiv. 10.1101/316471

[R34] LI Y. and KELLIS M. (2016). Joint Bayesian inference of risk variants and tissue-specific epigenomic enrichments across multiple complex human diseases. Nucleic Acids Research 44 e144. 27407109 10.1093/nar/gkw627 PMC5062982

[R35] LIU Q. and PIERCE D. A. (1994). A note on Gauss-Hermite quadrature. Biometrika 81 624–629.

[R36] MALLER J. B., MCVEAN G., BYRNES J., VUKCEVIC D., PALIN K., SU Z., HOWSON J. M. M., AUTON A., MYERS S., MORRIS A. (2012). Bayesian refinement of association signals for 14 loci in 3 common diseases. Nature Genetics 44 1294–1301. 23104008 10.1038/ng.2435 PMC3791416

[R37] MARENHOLZ I., GROSCHE S., KALB B., RÜSCHENDORF F., BLÜMCHEN K., SCHLAGS R., HARANDI N., PRICE M., HANSEN G., SEIDENBERG J., RÖBLITZ H., YÜREK S., TSCHIRNER S., HONG X., WANG X., HOMUTH G., SCHMIDT C. O., NÖTHEN M. M., HÜBNER N., NIGGEMANN B., BEYER K. and LEE Y.-A. (2017). Genome-wide association study identifies the SERPINB gene cluster as a susceptibility locus for food allergy. Nature Communications 8 1056. 10.1038/s41467-017-01220-0 PMC5648765 29051540

[R38] NAYLOR J. C. and SMITH A. F. (1982). Applications of a method for the efficient computation of posterior distributions. Journal of the Royal Statistical Society, Series C 31 214–225.

[R39] NEWCOMBE P. J., RAZA ALI H., BLOWS F. M., PROVENZANO E., PHAROAH P. D., CALDAS C. and RICHARDSON S. (2017). Weibull regression with Bayesian variable selection to identify prognostic tumour markers of breast cancer survival. Statistical Methods in Medical Research 26 414–436. 25193065 10.1177/0962280214548748 PMC6055985

[R40] NIKOOIENEJAD A., WANG W. and JOHNSON V. E. (2020). Bayesian variable selection for survival data using inverse moment priors. Annals of Applied Statistics 14 809. 33456641 10.1214/20-AOAS1325 PMC7808442

[R41] OJAVEE S. E., DARROUS L., PATXOT M., LÄLL K., FISCHER K., MÄGI R., KUTALIK Z. and ROBINSON M. R. (2023). Genetic insights into the age-specific biological mechanisms governing human ovarian aging. American Journal of Human Genetics 110 1549–1563. 37543033 10.1016/j.ajhg.2023.07.006 PMC10502738

[R42] PALMER C. N. A., IRVINE A. D., TERRON-KWIATKOWSKI A., ZHAO Y., LIAO H., LEE S. P., GOUDIE D. R., SANDILANDS A., CAMPBELL L. E., SMITH F. J. D., O’REGAN G. M., WATSON R. M., CECIL J. E., BALE S. J., COMPTON J. G., DIGIOVANNA J. J., FLECKMAN P., LEWIS-JONES S., ARSECULERATNE G., SERGEANT A., MUNRO C. S., EL HOUATE B., MCELREAVEY K., HALKJAER L. B., BISGAARD H., MUKHOPADHYAY S. and MCLEAN W. H. I. (2006). Common loss-of-function variants of the epidermal barrier protein filaggrin are a major predisposing factor for atopic dermatitis. Nature Genetics 38 441–446. 16550169 10.1038/ng1767

[R43] PEDERSEN E. M., AGERBO E., PLANA-RIPOLL O., STEINBACH J., KREBS M. D., HOUGAARD D. M., WERGE T., NORDENTOFT M., BoRGLUM A. D., MUSLINER K. L., GANNA A., SCHORK A. J., MORTENSEN P. B., MCGRATH J. J., PRIVÉ F. and VILHJÁLMSSON B. J. (2023). ADuLT: an efficient and robust time-to-event GWAS. Nature Communications 14 5553. 10.1038/s41467-023-41210-z PMC10492844 37689771

[R44] PIVIDORI M., SCHOETTLER N., NICOLAE D. L., OBER C. and IM H. K. (2019). Shared and distinct genetic risk factors for childhood-onset and adult-onset asthma: genome-wide and transcriptome-wide studies. Lancet Respiratory Medicine 7 509–522. 31036433 10.1016/S2213-2600(19)30055-4 PMC6534440

[R45] PRICE A. L., PATTERSON N. J., PLENGE R. M., WEINBLATT M. E., SHADICK N. A. and REICH D. (2006). Principal components analysis corrects for stratification in genome-wide association studies. Nature Genetics 38 904–909. 16862161 10.1038/ng1847

[R46] RAFTERY A. E., MADIGAN D. and HOETING J. A. (1997). Bayesian model averaging for linear regression models. Journal of the American Statistical Association 92 179–191.

[R47] SCHAID D. J., CHEN W. and LARSON N. B. (2018). From genome-wide associations to candidate causal variants by statistical fine-mapping. Nature Reviews Genetics 19 491–504. 10.1038/s41576-018-0016-z PMC6050137 29844615

[R48] SILLANPÄÄ M. J. and BHATTACHARJEE M. (2005). Bayesian association-based fine mapping in small chromosomal segments. Genetics 169 427–439. 15371355 10.1534/genetics.104.032680 PMC1448870

[R49] SINHA D., IBRAHIM J. G. and CHEN M.-H. (2003). A Bayesian justification of Cox’s partial likelihood. Biometrika 90 629–641.

[R50] SMITH F. J., IRVINE A. D., TERRON-KWIATKOWSKI A., SANDILANDS A., CAMPBELL L. E., ZHAO Y., LIAO H., EVANS A. T., GOUDIE D. R., LEWIS-JONES S. (2006). Loss-of-function mutations in the gene encoding filaggrin cause ichthyosis vulgaris. Nature Genetics 38 337–342. 16444271 10.1038/ng1743

[R51] SMYTH G. K. (2005). Numerical integration. Encyclopedia of Biostatistics 3088–3095.

[R52] STALEY J. R., JONES E., KAPTOGE S., BUTTERWORTH A. S., SWEETING M. J., WOOD A. M. and HOWSON J. M. (2017). A comparison of Cox and logistic regression for use in genome-wide association studies of cohort and case-cohort design. European Journal of Human Genetics 25 854–862. 28594416 10.1038/ejhg.2017.78 PMC5520083

[R53] SUDLOW C., GALLACHER J., ALLEN N., BERAL V., BURTON P., DANESH J., DOWNEY P., ELLIOTT P., GREEN J., LANDRAY M. (2015). UK Biobank: an open access resource for identifying the causes of a wide range of complex diseases of middle and old age. PLoS Medicine 12 e1001779. 25826379 10.1371/journal.pmed.1001779 PMC4380465

[R54] R CORE TEAM (2022). R: a language and environment for statistical computing. R Foundation for Statistical Computing, Vienna, Austria https://www.R-project.org/.

[R55] THERNEAU T. M. and GRAMBSCH P. M. (2000). Modeling survival data: extending the Cox model. Springer, New York, NY.

[R56] TOMIZUKA K., KOIDO M., SUZUKI A., YOSHINO S., TANAKA N., ISHIKAWA Y., LIU X., KOYAMA S., ISHIGAKI K., MURAKAWA Y., IMMUNE TRANSCRIPT/ENHANCER CONSORTIUM (ITEC), YAMAMOTO K. and TERAO C. (2024). Predicted constrained accessible regions mark regulatory elements and causal variants. bioRxiv. 10.1101/2024.10.31.621195

[R57] VALETTE K., LI Z., BON-BARET V., CHIGNON A., BÉRUBÉ J.-C., ESLAMI A., LAMOTHE J., GAUDREAULT N., JOUBERT P., OBEIDAT M., VAN DEN BERGE M., TIMENS W., SIN D. D., NICKLE D. C., HAO K., LABBÉ C., GODBOUT K., CÔTÉ A., LAVIOLETTE M., BOULET L.-P., MATHIEU P., THÉRIAULT S. and BOSSÉ Y. (2021). Prioritization of candidate causal genes for asthma in susceptibility loci derived from UK Biobank. Communications Biology 4 700. 34103634 10.1038/s42003-021-02227-6 PMC8187656

[R58] VICENTE C. T., REVEZ J. A. and FERREIRA M. A. R. (2017). Lessons from ten years of genome-wide association studies of asthma. Clinical and Translational Immunology 6 e165. 29333270 10.1038/cti.2017.54 PMC5750453

[R59] WAKEFIELD J. (2009). Bayes factors for genome-wide association studies: comparison with P-values. Genetic Epidemiology 33 79–86. 18642345 10.1002/gepi.20359

[R60] WALLACE C., CUTLER A. J., PONTIKOS N., PEKALSKI M. L., BURREN O. S., COOPER J. D., GARCÍA A. R., FERREIRA R. C., GUO H., WALKER N. M. (2015). Dissection of a complex disease susceptibility region using a Bayesian stochastic search approach to fine mapping. PLoS Genetics 11 e1005272. 26106896 10.1371/journal.pgen.1005272 PMC4481316

[R61] WANG G., SARKAR A., CARBONETTO P. and STEPHENS M. (2020). A simple new approach to variable selection in regression, with application to genetic fine mapping. Journal of the Royal Statistical Society, Series B 82 1273–1300. 10.1111/rssb.12388 PMC10201948 37220626

[R62] WEN X., LEE Y., LUCA F. and PIQUE-REGI R. (2016). Efficient integrative multi-SNP association analysis via deterministic approximation of posteriors. American Journal of Human Genetics 98 1114–1129. 27236919 10.1016/j.ajhg.2016.03.029 PMC4908152

[R63] YANG J., FERREIRA T., MORRIS A. P., MEDLAND S. E., MADDEN P. A. F., HEATH A. C., MARTIN N. G., MONTGOMERY G. W., WEEDON M. N., LOOS R. J. (2012). Conditional and joint multiple-SNP analysis of GWAS summary statistics identifies additional variants influencing complex traits. Nature Genetics 44 369–375. 22426310 10.1038/ng.2213 PMC3593158

[R64] YANG Y., TAYEB K., CARBONETTO P. and STEPHENS M. (2025). R code reproducing the analyses for “Bayesian variable selection in a Cox proportional hazards model with the ‘Sum of Single Effects’ prior”. 10.5281/zenodo.15391678

[R65] ZHONG X., MITCHELL R., BILLSTRAND C., THOMPSON E. E., SAKABE N. J., ANEAS I., SALAMONE I. M., GU J., SPERLING A. I., SCHOETTLER N., NÓBREGA M. A., HE X. and OBER C. (2025). Integration of functional genomics and statistical fine-mapping systematically characterizes adult-onset and childhood-onset asthma genetic associations. Genome Medicine 17 35. 40205616 10.1186/s13073-025-01459-z PMC11983851

[R66] ZHU Z., LEE P. H., CHAFFIN M. D., CHUNG W., LOH P.-R., LU Q., CHRISTIANI D. C. and LIANG L. (2018). A genome-wide cross-trait analysis from UK Biobank highlights the shared genetic architecture of asthma and allergic diseases. Nature Genetics 50 857–864. 29785011 10.1038/s41588-018-0121-0 PMC5980765

[R67] ZOU Y., CARBONETTO P., WANG G. and STEPHENS M. (2022). Fine-mapping from summary data with the “Sum of Single Effects” model. PLoS Genetics 18 e1010299. 35853082 10.1371/journal.pgen.1010299 PMC9337707

[R68] ZOU Y., CARBONETTO P., XIE D., WANG G. and STEPHENS M. (2023). Fast and flexible joint fine-mapping of multiple traits via the Sum of Single Effects model. bioRxiv. 10.1038/s41588-025-02486-7 PMC12900646 41634413

