# Supplementary material for: BAYESIAN VARIABLE SELECTION IN A COX PROPORTIONAL HAZARDS MODEL WITH THE “SUM OF SINGLE EFFECTS” PRIOR
Source: ArXiv. 2025 Jun 6:arXiv:2506.06233v1. Preprint. [Version 1] (PMC12155538)
Supplement: Supplement 1 [file NIHPP2506.06233v1-supplement-1.pdf]

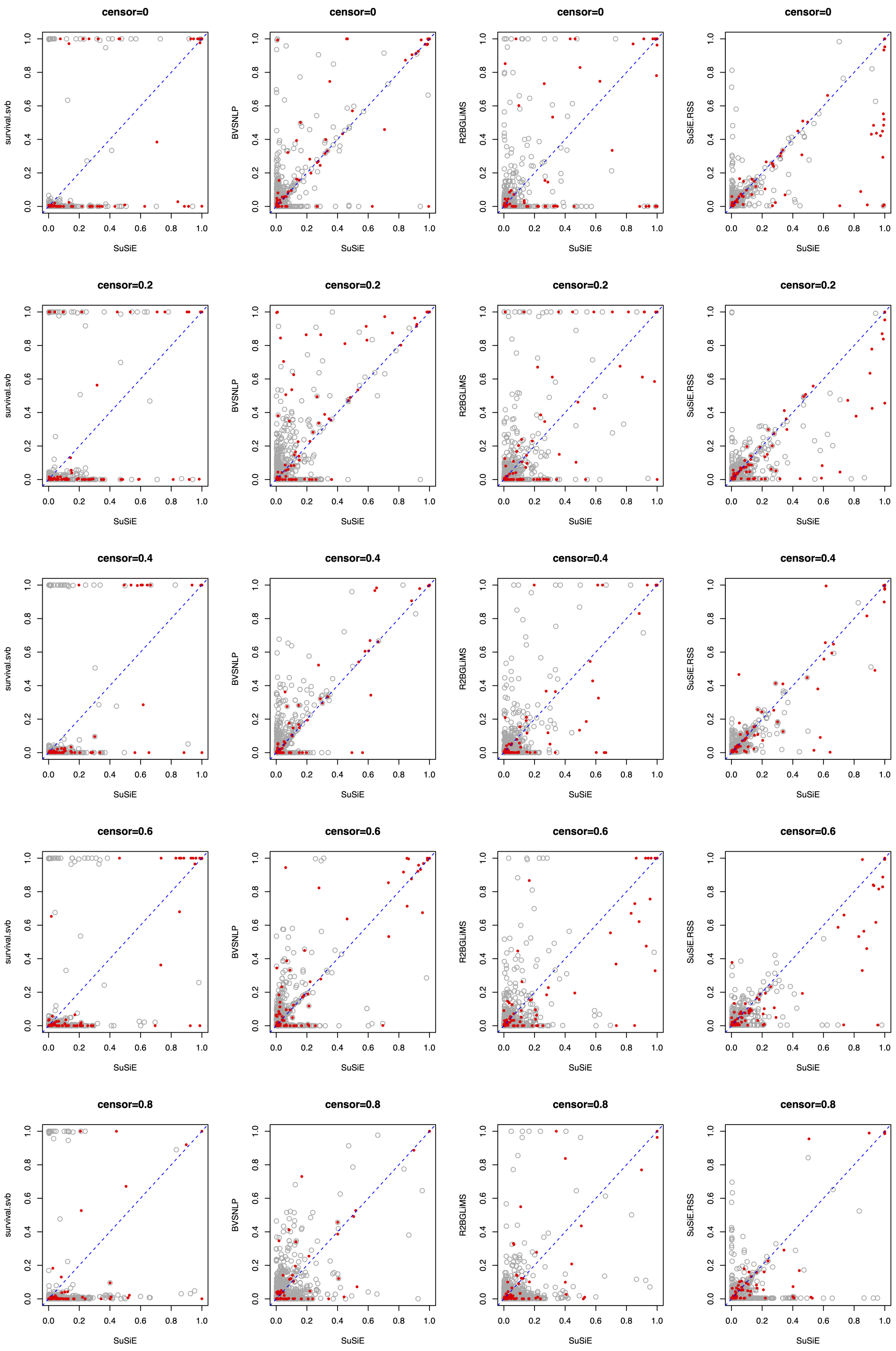

SUPPLEMENTARY FIGURE 1. CoxPH-SuSiE PIPs (x-axis) vs. PIPs from other methods (y-axis) in the GTEx simulations, separately at different censoring levels. Each point is single SNP; causal SNPs are shown as solid red circles, and other SNPs are shown as open gray circles.

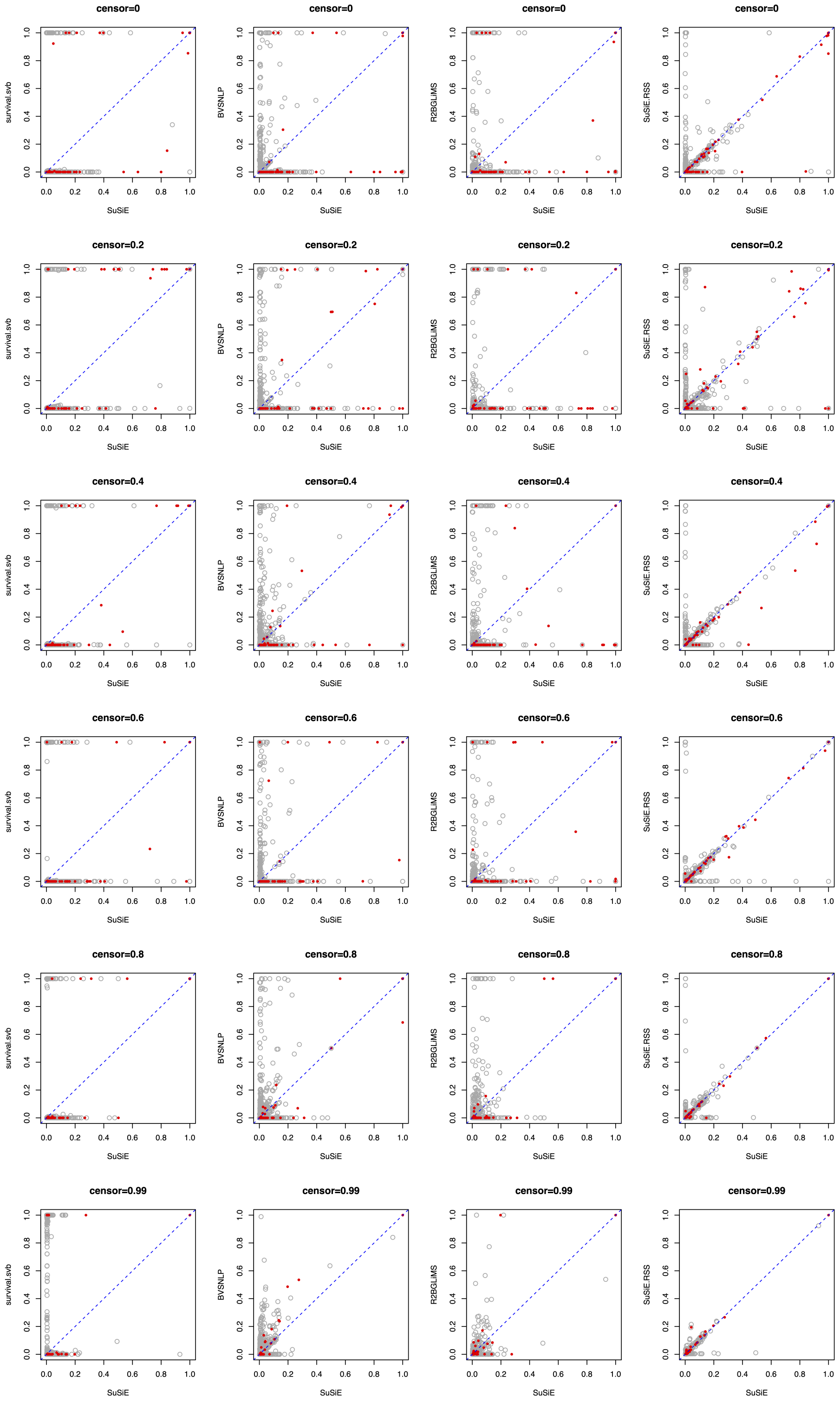

SUPPLEMENTARY FIGURE 2. CoxPH-SuSiE PIPs (x-axis) vs. PIPs from other methods (y-axis) in the UK Biobank simulations, separately at different censoring levels. Each point is a single SNP; causal SNPs are shown as solid red circles, and other SNPs are shown as open gray circles.

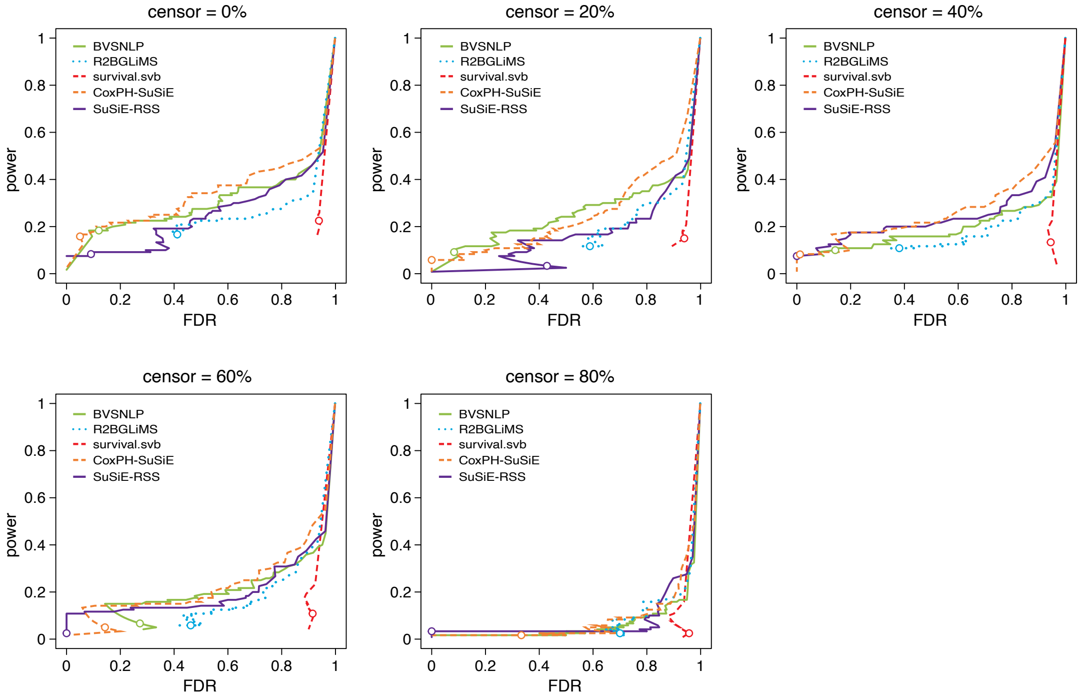

SUPPLEMENTARY FIGURE 3. Discovery of causal SNPs using PIPs in the GTEx simulations. Each curve shows power vs. FDR in identifying causal SNPs. For each power-vs-FDR plot, the FDR and power were calculated from the results on 80 data sets as the PIP threshold was varied from 0 to 1. Open circles are drawn at a PIP threshold of 0.95.

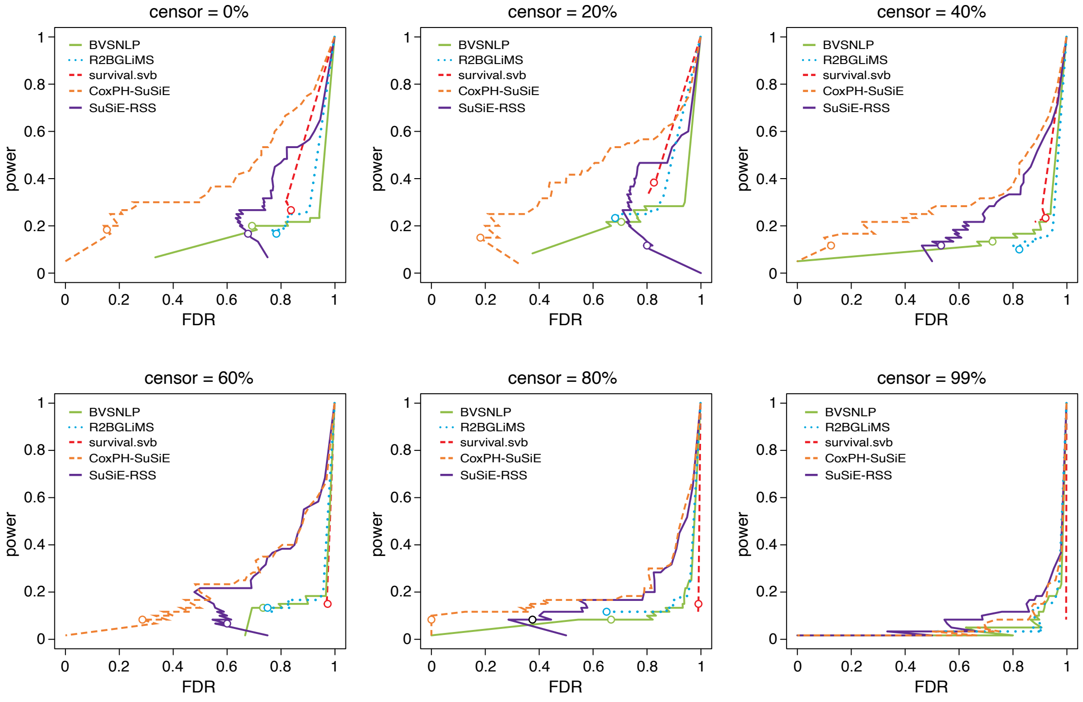

SUPPLEMENTARY FIGURE 4. Discovery of causal SNPs using PIPs in the UK Biobank simulations. Each curve shows power vs. FDR in identifying causal SNPs. For each power-vs-FDR plot, FDR and power were calculated from the results on 40 data sets as the PIP threshold was varied from 0 to 1. Open circles are drawn at a PIP threshold of 0.95.
